# Evaluation of Field Sobriety Tests for Identifying Drivers Under the Influence of Cannabis: A Randomized Clinical Trial

**DOI:** 10.1001/jamapsychiatry.2023.2345

**Published:** 2023-08-02

**Authors:** Thomas D. Marcotte, Anya Umlauf, David J. Grelotti, Emily G. Sones, Kyle F. Mastropietro, Raymond T. Suhandynata, Marilyn A. Huestis, Igor Grant, Robert L. Fitzgerald

**Affiliations:** 1 Center for Medicinal Cannabis Research, Department of Psychiatry, University of California, San Diego; 2 Joint Doctoral Program in Clinical Psychology, San Diego State University/University of California, San Diego; 3 Center for Medicinal Cannabis Research, Department of Pathology, University of California, San Diego; 4 Center for Medicinal Cannabis Research, Skaggs School of Pharmacy and Pharmaceutical Sciences, University of California, San Diego; 5 Institute for Emerging Health Professions, Thomas Jefferson University, Philadelphia, Pennsylvania

## Abstract

**Importance:**

With increasing medicinal and recreational cannabis legalization, there is a public health need for effective and unbiased evaluations for determining whether a driver is impaired due to Δ^9^-tetrahydrocannabinol (THC) exposure. Field sobriety tests (FSTs) are a key component of the gold standard law enforcement officer–based evaluations, yet controlled studies are inconclusive regarding their efficacy in detecting whether a person is under the influence of THC.

**Objective:**

To examine the classification accuracy of FSTs with respect to cannabis exposure and driving impairment (as determined via a driving simulation).

**Design, Setting, and Participants:**

This double-blind, placebo-controlled parallel randomized clinical trial was conducted from February 2017 to June 2019 at the Center for Medicinal Cannabis Research, University of California, San Diego. Participants were aged 21 to 55 years and had used cannabis in the past month. Data were analyzed from August 2021 to April 2023.

**Intervention:**

Participants were randomized 1:1:1 to placebo (0.02% THC), 5.9% THC cannabis, or 13.4% THC cannabis smoked ad libitum.

**Main Outcome and Measures:**

The primary end point was law enforcement officer determination of FST impairment at 4 time points after smoking. Additional measures included officer estimation as to whether participants were in the THC or placebo group as well as driving simulator data. Officers did not observe driving performance.

**Results:**

The study included 184 participants (117 [63.6%] male; mean [SD] age, 30 [8.3] years) who had used cannabis a mean (SD) of 16.7 (9.8) days in the past 30 days; 121 received THC and 63, placebo. Officers classified 98 participants (81.0%) in the THC group and 31 (49.2%) in the placebo group as FST impaired (difference, 31.8 percentage points; 95% CI, 16.4-47.2 percentage points; *P* < .001) at 70 minutes after smoking. The THC group performed significantly worse than the placebo group on 8 of 27 individual FST components (29.6%) and all FST summary scores. However, the placebo group did not complete a median of 8 (IQR, 5-11) FST components as instructed. Of 128 participants classified as FST impaired, officers suspected 127 (99.2%) as having received THC. Driving simulator performance was significantly associated with results of select FSTs (eg, ≥2 clues on One Leg Stand was associated with impairment on the simulator: odds ratio, 3.09; 95% CI, 1.63-5.88; *P* < .001).

**Conclusions and Relevance:**

This randomized clinical trial found that when administered by highly trained officers, FSTs differentiated between individuals receiving THC vs placebo and driving abilities were associated with results of some FSTs. However, the high rate at which the participants receiving placebo failed to adequately perform FSTs and the high frequency that poor FST performance was suspected to be due to THC-related impairment suggest that FSTs, absent other indicators, may be insufficient to denote THC-specific impairment in drivers.

**Trial Registration:**

ClinicalTrials.gov Identifier: NCT02849587

## Key Points

**Question:**

How accurate are field sobriety tests (FSTs) in identifying acute Δ^9^-tetrahydrocannabinol (THC) impairment?

**Findings:**

In this randomized clinical trial of 184 cannabis users randomized to THC or placebo, law enforcement officers classified 81.0% and 49.2%, respectively, as FST impaired, and officers suspected that 99.2% of FST-impaired participants received THC. Driving simulator performance was associated with select FSTs.

**Meaning:**

In this study, FSTs differentiated between THC- and placebo-exposed participants; however, the substantial overlap of FST impairment between groups and the high frequency at which FST impairment was suspected to be due to THC suggest that absent other indicators, FSTs alone may be insufficient to identify THC-specific driving impairment.

## Teaser

This randomized clinical trial investigates the accuracy of field sobriety tests administered by law enforcement officers to assess functional impairment and driving performance among individuals who have smoked cannabis.

## Introduction

Cannabis use and legalization have expanded in recent decades.^1^ In addition to recreational purposes, cannabis is often used by the public to treat medical and psychiatric symptoms, including anxiety, insomnia, chronic pain, and depression.^2^

The risk of impaired driving due to Δ^9^-tetrahydrocannabinol (THC) is a significant concern since THC exposure has been associated with worse cognition and psychomotor functioning.^3^ However, individual responses to THC vary,^4^ and not all individuals show significant declines in driving performance.^5^ Unlike the association of blood alcohol concentrations with impairment,^6^ THC blood concentrations do not correlate with driving performance,^5^ likely reflecting its unique pharmacokinetics (eg, rapid distribution to various tissue compartments).^7^ In addition, in regular users, THC is detectable many hours to days after use,^8^ long after impairment wanes.

The legal standard for driving impairment is when an individual’s “mental or physical abilities are so impaired that he or she is no longer able to drive a vehicle with the caution of a sober person, using ordinary care, under similar circumstances.”^9^ In the field, impairment is determined via observation of driving behavior (ie, vehicle in motion), driver interviews, and field sobriety tests (FSTs), which examine abilities, such as balance, coordination, divided attention, and eye movements. Select FSTs have been validated based on alcohol ingestion.^10,11,12^

Studies of FSTs conducted after cannabis exposure have reached disparate conclusions,^13^ finding mild to moderate^14,15,16,17^ or little to no^18,19^ sensitivity to THC, likely due to different THC doses, administration methods, testing times after administration, and participant characteristics. One court concluded that “there is as yet no scientific agreement on whether, and, if so, to what extent, these types of tests are indicative of marijuana intoxication.”^20^

In controlled studies, many previous study evaluations were conducted by research staff (or not delineated) rather than trained officers^16,18,19,21^ and participants were exposed to FSTs prior to drug administration.^15,18^ Both may reduce test sensitivity and increase the likelihood of individuals receiving placebo doing well on the FSTs. Sample sizes were typically small, with outcomes often based on drug exposure rather than driving impairment.^15,18,21^

Given the aforementioned issues and the increased appreciation of confirmation and unconscious biases,^22^ including within policing,^23,24^ validation of objective, unbiased, and effective methods for discriminating between drivers who are or are not impaired by cannabis is critical in ensuring equitable enforcement of driving-under-the-influence laws. The aim of this study was to examine the classification accuracy of law enforcement officer–administered FSTs to assess (1) cannabis exposure and (2) driving simulator impairment.

## Methods

This randomized clinical trial was conducted from February 2017 to June 2019 at the Center for Medicinal Cannabis Research, University of California, San Diego (NCT02849587; trial protocol in Supplement 1). The trial was approved by the University of California, San Diego institutional review board; the US Food and Drug Administration; and the Research Advisory Panel of California and was conducted in accordance with the Declaration of Helsinki.^25^ Participants provided written informed consent. We followed the Consolidated Standards of Reporting Trials (CONSORT) reporting guideline.

### Participants

Participants were recruited via community outreach and ClinicalTrials.gov. Inclusion criteria were age 21 to 55 years, cannabis use 4 or more times in the past month, holding a valid driver’s license, and driving at least 1000 miles in the past year. Exclusion criteria were history of traumatic brain injury; significant medical conditions or psychiatric conditions; positive pregnancy test result; urine screen positive for nonprescription amphetamines, benzodiazepines, barbiturates, opiates, oxycodone, or cocaine, methamphetamine, or phencyclidine; past-year substance use disorder; and oral fluid THC level higher than 5 ng/mL on the testing day. Participants’ race and ethnicity were ascertained by participant self-report and to show the representativeness of the groups; categories included African American, Asian, Hispanic, Indigenous, multiracial, non-Hispanic White, and unknown.

### Study Design

This study was part of a double-blind, placebo-controlled, parallel clinical trial.^5^ Participants were randomized 1:1:1 using permuted blocks stratified by prior cannabis exposure (using cannabis ≥4 times per week or <4 times per week in the past month) to smoke a cannabis cigarette with either 13.4%, 5.9%, or 0.02% (placebo) THC content. They were to abstain from cannabis for at least 2 days prior to the training and experiment days. On the experiment day, participants completed a urine drug screen and breathalyzer for alcohol and had an oral fluid sample obtained for point-of-collection THC detection (Dräger DrugTest 5000) (final determination was via liquid chromatography tandem mass spectrometry^26^).

Participants completed a driving simulation and had a biosample collected prior to smoking and at postsmoking time points. Simulator^5^ and toxicology^26,27^ results were reported previously.

### FSTs

The Walk and Turn (WAT), One Leg Stand (OLS), Finger to Nose (FTN), Lack of Convergence (LOC), and Modified Romberg (MROM) tests were administered a median of 1 hour 10 minutes, 2 hours 20 minutes, 3 hours 10 minutes, and 4 hours 10 minutes after smoking THC (eTable 1 in Supplement 2). The WAT and OLS tests, along with horizontal gaze nystagmus, constitute the standardized field sobriety tests (SFSTs) based on validation with alcohol. Horizontal gaze nystagmus was not assessed due to drug recognition expert (DRE) input that it is unlikely to be affected by THC and to time limitations.

*Clue* is the term used by law enforcement officers when an individual does not adequately perform a component of the FST and is an indicator of FST impairment.^10^ Two or more clues on the WAT or OLS test discriminate between individuals with blood alcohol concentrations above and below 0.08%^12^; impairment on 2 or more tests suggests overall FST impairment. In the current study, officers determined FST impairment based on performance across all FSTs. We refer to FST impairment since final law enforcement determination of impairment includes driving behavior and interviews.

Law enforcement officers were asked, “Which treatment do you think the participant received?” Answers were given using a 5-point scale (from “strongly believed…real marijuana” [1] to “strongly believed…placebo” [5]).

Field sobriety tests were administered by certified DRE instructors (n = 11), the highest training level for impaired driving detection, from the California DRE program. One officer evaluated the participant at all time points and was blinded to treatment assignment.

### Driving Simulations

Driving simulations occurred at approximately 30 minutes, 1 hour 30 minutes, 3 hours 30 minutes, and 4 hours 30 minutes after smoking. Simulations (approximately 25 minutes) were presented on a STISIM M300WS-Console Driving Simulator System (Systems Technology, Inc) consisting of 3 screens with wide field-of-view monitors, a steering wheel, an accelerator, and a brake pedal.^5^ A composite drive score, composed of key driving variables, represented global driving performance (eAppendix in Supplement 2). Officers did not observe simulator performance since the goal of the study was to determine the degree to which FSTs yield impairment determinations in the absence of factors that might increase the risk of confirmation bias.

### Study Drug Administration

Cannabis from the National Institute on Drug Abuse Drug Supply Program containing 5.9% THC, 13.4% THC, or placebo (0.02% THC) was hand-rolled into 700-mg cigarettes. Participants were instructed, “Smoke the cigarette the way you do at home to get high. You may take up to 10 minutes.” They were to smoke ad libitum, with a minimum of 4 puffs required.

### Statistical Analysis

Analyses were conducted from August 2021 to April 2023 with R, version 4.2.0 (R Project for Statistical Computing). Tests were 2-sided with a significance level of *P* < .05. Group comparisons used 2-sample *t* tests and χ^2^ tests or their nonparametric alternatives. Binary outcomes (THC exposure, FST impairment classification, and driving simulator impairment) were tested using logistic regression methods, including Firth penalized-likelihood regression. Analyses involving FSTs were corrected for multiple testing using the false discovery rate method to keep the familywise type I error to 0.05. False discovery rate adjustments were applied to *P* values and 95% CIs when analyzing the clues within each FST and separately for summarized clues. Both unadjusted and adjusted results are reported.

The 2 THC arms (5.9% and 13.4%) were combined based on prior results showing no statistical or practical differences in driving performance.^5^ Proportions of FST impairment at the 4 time points were compared among the 3 arms, showing differences between placebo and both THC groups but not between the THC groups.

## Results

**Figure 1.**
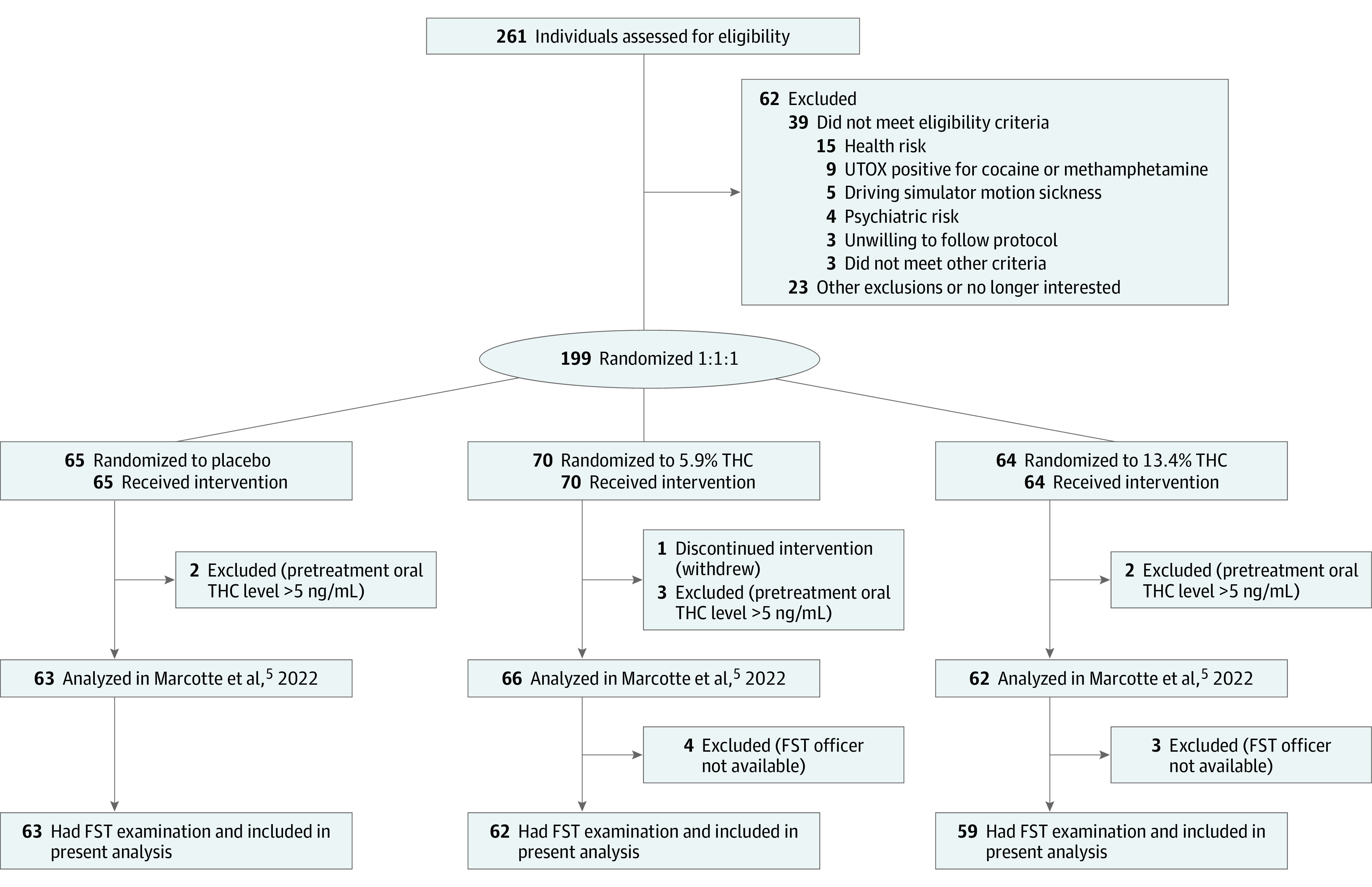
CONSORT Diagram Showing Participant Inclusion and Exclusion From Initial Screening to Final Sample FST indicates field sobriety test; THC, Δ^9^-tetrahydrocannabinol and UTOX, urine toxicology screen.

Of 261 individuals screened (Figure 1), 199 were randomized. Seven were excluded due to presmoking oral fluid THC levels higher than 5 ng/mL, and 1 withdrew after smoking; officers were unavailable for 7 participants. The final sample included 184 participants, of whom 67 (36.4%) were female and 117 (63.6%) were male; mean (SD) age was 30 (8.3) years. A total of 17 individuals (9.2%) were African American; 16 (8.7%), Asian; 55 (29.9%), Hispanic; 8 (4.3%), Indigenous; 80 (43.5%) non-Hispanic White; and 6 (3.3%), multiracial; 2 (1.1%) had unknown race and ethnicity. Participants had used cannabis a mean (SD) of 16.7 (9.8) days in the past 30 days. The placebo (63 participants [34.2%]) and THC (121 participants [65.8%]) groups were similar in background characteristics, although the placebo group was younger, with greater female representation (Table 1). Median self-reported highness (scale of 0 to 100, with higher scores indicating more impairment) at 30 minutes was 64 (IQR, 32-76) for the THC group and 13 (IQR, 1-28) for the placebo group (*P* < .001).

**Table 1.** Descriptive Statistics of Study Participants

Variable	Participants^a^	
	Placebo (n = 63)	5.9% or 13.4% THC (n = 121)
Age, mean (SD), y	28.1 (7.25)	31.0 (8.65)
Sex		
Female	31 (49.2)	36 (29.8)
Male	32 (50.8)	85 (70.2)
Educational level, mean (SD), y	15.0 (1.93)	15.0 (2.04)
Race and ethnicity		
African American	8 (12.7)	9 (7.4)
Asian	5 (7.9)	11 (9.1)
Hispanic	15 (23.8)	40 (33.1)
Indigenous	5 (7.9)	3 (2.5)
Non-Hispanic White	28 (44.4)	52 (43.0)
Multiracial	2 (3.2)	4 (3.3)
Unknown	0	2 (1.7)
Distance driven in previous year, median (IQR), mi	8730 (5420-12 825)	8960 (5048-13 290)
Cannabis use		
Current use <4 times/wk	34 (54.0)	62 (51.2)
Days used in past 30 d, mean (SD), No.	16.9 (9.69)	16.6 (9.94)
Amount used in past 30 d, median (IQR), g/d	0.55 (0.25-1.00)	0.55 (0.25-1.00)
Time since last use, median (IQR), d	3.00 (3.00-6.00)	3.00 (2.50-4.00)

Abbreviation: THC, Δ^9^-tetrahydrocannabinol.

^a^ Data are presented as the number (percentage) of participants unless otherwise indicated.

### FST Performance and Officer Determinations of THC vs Placebo

**Figure 2.**
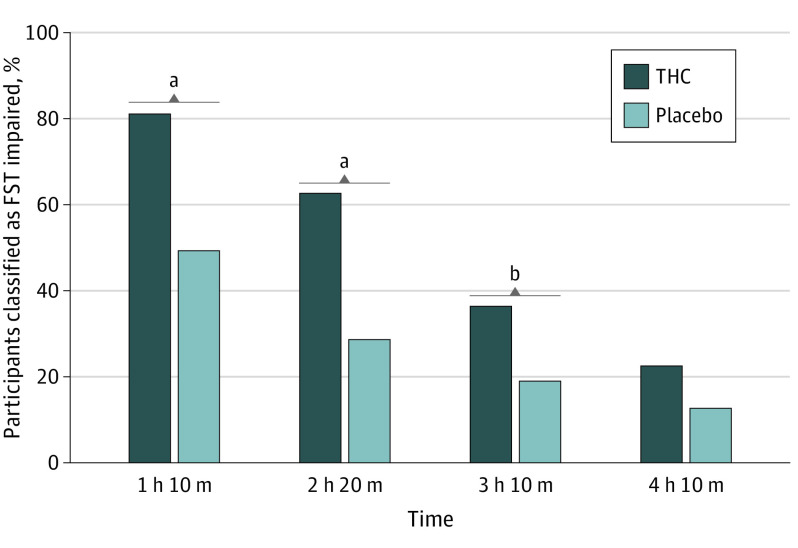
Officer Classifications of Field Sobriety Test (FST) Impairment for the Δ^9^-Tetrahydrocannabinol (THC) and Placebo Groups Over the 4 Time Points ^a^*P* < .001. ^b^*P* < .05.

Officers classified 98 participants (81.0%) in the THC group and 31 (49.2%) in the placebo group as FST impaired at the first evaluation (difference, 31.8 percentage points; 95% CI, 16.4-47.2 percentage points; *P* < .001) (Figure 2) (ie, sensitivity of 81.0% [n = 98] and specificity of 50.8% [n = 32] to THC exposure). Officers suspected that 86.0% (n = 104) of the THC group and 54.0% (n = 34) of the placebo group received active THC; they were uncertain for 5 participants (3 placebo [4.8%], 2 THC [1.7%]).

Of the 96 participants who officers strongly believed received THC, 95 (99.0%) were classified as FST impaired; none of the 23 participants who were strongly believed to have received placebo were classified as FST impaired (eTable 2 in Supplement 2). Officers somewhat believed that 41 of the 182 participants (22.5%) received THC (32 of those [78.0%] were classified FST impaired) and that 17 of 182 (9.3%) received placebo (1 of those [5.9%] was classified as FST impaired). When strongly and somewhat were combined, 127 of the 137 participants (92.7%) who were believed to have received THC were classified as FST impaired; 1 of 40 participants (2.5%) who the officers believed received placebo was classified as FST impaired. Of all participants classified as FST impaired (n = 128), officers believed strongly (95 participants [74.2%]) or somewhat (32 participants [25.0%]) that the participant received THC (127 total [99.2%]) (eTable 3 in Supplement 2).

The THC group had a substantially higher percentage of participants with FST clues (failing to adequately perform on a component) compared with the placebo group overall (8 of 27 [29.6%]) and specifically on the WAT, OLS, FTN, and LOC tests as well as a higher median number of total clues (11 [IQR, 9-13] vs 8 [IQR, 5-11]), but between-group differences were not found for the MROM test (Table 2). The THC group also performed significantly worse than the placebo group on all FST summary scores (except MROM). A higher proportion of the THC group than the placebo group exceeded SFST cut points validated for alcohol (≥2 clues) for the WAT test (92 [76.0%] vs 35 [56.5%]; *P* = .007) and OLS test (69 [58.5%] vs 23 [37.1%]; *P* = .007) individually and had more than 2 clues on both WAT and OLS (56 [47.5%] vs 17 [27.9%]; *P* = .01).

**Table 2.** Univariable Analysis of the Association Between FST Items and THC Exposure

SFST clue	Participants with clues^a^		Unadjusted analysis		Adjusted analysis^b^	
	Placebo (n = 63)	THC (n = 121)	OR (95% CI)	*P* value	95% CI	*P* value
Walk and Turn Test						
Instructions	13 (20.6)	36 (30.0)^c^	1.65 (0.80-3.40)	.18	0.76-3.58	.25
Balance	17 (27.0)	56 (46.3)	2.33 (1.20-4.52)	.01	1.04-5.23	.04
Starts too soon	3 (4.8)^d^	10 (8.3)	1.77 (0.47-6.69)	.40	0.45-6.92	.45
Stops when walking	20 (32.3)^d^	58 (47.9)	1.93 (1.02-3.67)	.04	0.92-4.08	.10
Steps off line	8 (12.9)^d^	38 (31.4)	3.09 (1.34-7.13)	.008	1.05-9.11	.04
Wrong number of steps	8 (12.9)^d^	29 (24.0)	2.13 (0.91-4.99)	.08	0.82-5.54	.15
Misses heel to toe	9 (14.5)^d^	43 (35.5)	3.25 (1.46-7.22)	.004	1.05-10.1	.03
Raises arm to balance	28 (45.2)^d^	67 (55.4)	1.51 (0.81-2.79)	.19	0.79-2.88	.25
Improper turn	29 (46.8)^d^	60 (49.6)	1.12 (0.61-2.07)	.72	0.61-2.07	.72
Total clues, median (IQR)	2.0 (1.0-3.0)	3.0 (2.0-4.0)	1.52 (1.22-1.88)	<.001	1.17-1.98	<.001
≥2 WAT clues	35 (56.5)^d^	92 (76.0)	2.45 (1.27-4.70)	.007	NA	NA
One Leg Stand Test						
Puts foot down	10 (16.1)^d^	43 (35.8)^c^	2.90 (1.34-6.29)	.007	1.20-7.03	.01
Uses arms to balance	21 (33.9)^d^	57 (47.5)^c^	1.77 (0.93-3.34)	.08	0.90-3.47	.11
Sways	35 (55.6)	102 (84.3)	4.29 (2.14-8.63)	<.001	1.77-10.5	<.001
Hops	2 (3.2)^d^	10 (8.5)^e^	2.78 (0.59-13.1)	.20	0.59-13.1	.20
Total clues, median (IQR)	1.0 (0.0-2.0)	2.0 (1.0-2.75)	1.84 (1.34-2.54)	<.001	1.26-2.69	<.001
≥2 OLS clues	23 (37.1)^d^	69 (58.5)^e^	2.39 (1.27-4.49)	.007	NA	NA
≥2 WAT clues and ≥2 OLS clues	17 (27.9)^d^^,^^f^	56 (47.5)^e^	2.34 (1.20-4.55)	.01	NA	NA
Finger to Nose Test						
Instructions	13 (20.6)	39 (32.2)	1.83 (0.89-3.76)	.10	0.79-4.26	.23
Incorrect sequence	9 (14.3)	21 (17.4)	1.26 (0.54-2.94)	.59	0.52-3.03	.69
Uses pad rather than finger	32 (50.8)	74 (61.2)	1.53 (0.83-2.82)	.18	0.77-3.03	.31
Leaves finger on nose	8 (12.7)	15 (12.4)	0.97 (0.39-2.44)	.95	0.39-2.44	.95
Eyelid tremor	45 (71.4)	91 (75.2)	1.21 (0.61-2.41)	.58	0.60-2.47	.69
Body tremor	6 (9.5)	35 (28.9)	3.87 (1.53-9.78)	.004	1.21-12.3	.02
Sways	22 (34.9)	77 (64.2)^c^	3.34 (1.76-6.32)	<.001	1.39-8.02	.002
Total clues, median (IQR)	2.0 (1.0-3.0)	3.0 (2.0-4.0)	1.54 (1.21-1.95)	.001	1.18-2.00	.001
Lack of Convergence						
Instructions	3 (4.8)	10 (8.3)	1.80 (0.48-6.80)	.38	0.48-6.80	.38
Eyes do not converge	31 (49.2)	83 (68.6)	2.25 (1.21-4.21)	.01	1.10-4.61	.02
Pupillary diameter, median (IQR), mm^g^	5.0 (4.0-6.0)	5.5 (4.5-6.0)	1.31 (0.99-1.74)	.06	NA	NA
Total clues, median (IQR)	1.0 (0.0-1.0)	1.0 (0.0-1.0)	2.31 (1.27-4.20)	.006	1.24-4.32	.007
Modified Romberg Balance Test						
Instructions	20 (31.7)	35 (29.2)^c^	0.89 (0.46-1.71)	.72	0.44-1.79	>.99
Internal clock, median (IQR), s^h^	34 (30-42)	32 (29-36)	0.96 (0.94-0.99)	.004	NA	NA
Internal clock not acceptable	34 (54.0)	49 (40.8)^c^	0.59 (0.32-1.09)	.09	0.26-1.32	.45
Eyelid tremors	50 (79.4)	96 (79.3)	1.00 (0.47-2.12)	>.99	0.47-2.12	>.99
Body tremors	17 (27.0)	32 (26.4)	0.97 (0.49-1.93)	.94	0.48-1.95	>.99
Sways	40 (63.5)	87 (73.1)^i^	1.56 (0.81-3.01)	.18	0.72-3.40	.45
Total clues, median, (IQR)	2.0 (2.0-3.0)	2.0 (2.0-3.0)	0.93 (0.70-1.24)	.63	0.70-1.24	.63
Total clues for all tests, median (IQR)	8.0 (5.0-11.0)	11.0 (9.0-13.0)	1.23 (1.12-1.35)	<.001	1.12-1.35	<.001

Abbreviations: FST, field sobriety test; NA, not applicable; OLS, One Leg Stand; OR, odds ratio; SFST, standardized field sobriety test; THC, Δ^9^-tetrahydrocannabinol; WAT, Walk and Turn.

^a^ Data are presented as the number (percentage) of participants who showed each clue unless otherwise indicated.

^b^ Adjusted 95% CIs and *P* values are based on the false discovery rate method.

^c^ n = 120.

^d^ n = 62.

^e^ n = 118.

^f^ n = 61.

^g^ Numeric variable (not included in the calculation of total clues).

^h^ Numeric variable for the participant’s estimate of when 30 seconds had passed (not included in the calculation of total clues).

^i^ n = 119.

When asked to estimate when 30 seconds had passed (MROM), the THC group was closer to the correct time (median, 32 seconds [IQR, 29-36 seconds]) compared with the placebo group (median, 34 seconds [IQR, 30-42 seconds]) (*P* = .004). Pupillary size (LOC test) at approximately 70 minutes after smoking was not significantly different between groups (THC: median, 5.5 mm [IQR, 4.5-6.0 mm]; placebo: 5.0 mm [IQR, 4.0-6.0 mm]; *P* = .06).

### FST Impairment Time Course

Declining percentages of both groups were classified as FST impaired at subsequent evaluations (Figure 2). Slopes did not differ between groups (χ^2^_3_ = 4.82; *P* = .18). The THC group showed significant improvement in total clues after adjusting for practice effects, decreasing from 10.8 to 9.2 clues (*P* < .001).^28^ The 2 groups’ score changes significantly differed after this adjustment (decrease of 1.56 in the THC group compared with 0.05 in the placebo group; *P* = .007), suggesting recovery of functioning in the THC group. Improvement was observed on several FSTs; rates did not differ between groups for any clue (eTable 4 in Supplement 2).

### FST Impairment vs Nonimpairment in the Placebo Group

There were no differences in demographics, cannabis use history, treatment guess, blood THC concentration, or composite drive scores between placebo participants classified as FST impaired or unimpaired (eTable 5 in Supplement 2). Participants classified as FST impaired had more FST clues (eTable 6 in Supplement 2).

### FSTs and Driving Simulator Performance

Impaired simulator performance was associated with worse FST performance. Significantly higher odds ratios for clues ranged from 2.00 (95% CI, 1.00-4.00) to 3.03 (95% CI, 1.62-5.68) on WAT, from 2.17 (95% CI, 1.57-3.02) to 5.52 (95% CI, 2.19-13.9) on OLS, and from 1.32 (95% CI, 1.05-1.66) to 2.86 (95% CI, 1.39-5.87) on FTN (Table 3). The LOC and MROM tests were not associated with simulator performance. Worse simulator performance in the THC group was uniquely associated with clues on the WAT, OLS, and FTN tests and with total clues (Table 3). Overall, FST impairment had a sensitivity of 80.9% and specificity of 35.7% to driving simulator impairment.

**Table 3.** Percentage of Participants Impaired or Not Impaired on the Driving Simulator Who Exhibited Clues on FSTs at First FST Evaluation

SFST clue	Participants with clues^a^		Unadjusted analysis^b^		Adjusted analysis^c^	
	Not impaired on simulator (n = 112)	Impaired on simulator (n = 68)	Odds ratio (95% CI)	*P* value	95% CI	*P* value
Walk and Turn Test						
Instructions	23 (20.5)	24 (35.8)^d^	2.16 (1.10-4.25)	.03	1.01-4.62	.05
Balance^e^	33 (29.5)	38 (55.9)	3.03 (1.62-5.68)	.001	1.25-7.37	.005
Starts too soon	9 (8.0)	4 (6.0)^d^	0.73 (0.21-2.46)	.61	0.21-2.46	.61
Stops when walking	45 (40.2)	30 (44.8)^d^	1.21 (0.65-2.23)	.55	0.65-2.26	.61
Steps off line	22 (19.6)	22 (32.8)^c^	2.00 (1.00-3.99)	.05	0.94-4.23	.07
Wrong number of steps^e^	17 (15.2)	20 (29.9)^d^	2.38 (1.14-4.96)	.02	1.01-5.60	.05
Misses heel to toe^e^	24 (21.4)	27 (40.3)^d^	2.47 (1.27-4.81)	.008	1.05-5.86	.03
Raises arm to balance	51 (45.5)	40 (59.7)^d^	1.77 (0.96-3.27)	.07	0.93-3.38	.09
Improper turn	47 (42.0)	40 (59.7)^d^	2.05 (1.11-3.79)	.02	1.01-4.16	.05
Total clues, median (IQR)^e^	2.0 (1.0-3.0)	3.0 (2.0-5.0)	1.50 (1.23-1.83)	<.001	1.18-1.90	<.001
≥2 WAT clues	71 (63.4)	52 (77.6)^d^	2.00 (1.00-4.00)	.05	NA	NA
One Leg Stand Test						
Puts foot down^e^	22 (20.0)^d^	31 (45.6)	3.35 (1.72-6.54)	<.001	1.56-7.19	.001
Uses arms to balance	37 (33.3)^f^	38 (56.7)^d^	2.62 (1.40-4.89)	.002	1.35-5.08	.003
Sways^e^	73 (65.2)	62 (91.2)	5.52 (2.19-13.9)	<.001	1.82-16.7	.001
Hops	4 (3.6)^g^	8 (12.1)^h^	3.66 (1.06-12.7)	.04	1.06-12.7	.04
Total clues, median (IQR)	1.0 (0.0-2.0)	2.0 (1.0-3.0)	2.17 (1.57-3.02)	<.001	1.41-3.35	<.001
≥2 OLS clues	45 (40.9)^g^	45 (68.2)^h^	3.09 (1.63-5.88)	<.001	NA	NA
≥2 WAT clues and ≥2 OLS clues	35 (31.8)^g^	36 (55.4)^i^	2.66 (1.41-5.01)	.002	NA	NA
Finger to Nose Test						
Instructions	26 (23.2)	26 (38.2)	2.05 (1.06-3.95)	.03	0.93-4.53	.09
Incorrect sequence	19 (17.0)	11 (16.2)	0.94 (0.42-2.13)	.89	0.42-2.13	.89
Uses pad rather than finger	62 (55.4)	42 (61.8)	1.30 (0.70-2.41)	.40	0.67-2.52	.56
Leaves finger on nose	14 (12.5)	9 (13.2)	1.07 (0.44-2.62)	.89	0.43-2.62	.89
Eyelid tremor	86 (76.8)	48 (70.6)	0.73 (0.37-1.43)	.36	0.34-1.53	.56
Body tremor^e^	17 (15.2)	23 (33.8)	2.86 (1.39-5.87)	.004	1.06-7.68	.03
Sways	54 (48.2)	43 (64.2)^d^	1.92 (1.03-3.58)	.04	0.93-3.99	.09
Total clues, median (IQR)	3.0 (2.0-3.0)	3.0 (2.0-4.0)	1.32 (1.05-1.66)	.02	1.03-1.70	.03
Lack of Convergence						
Instructions	7 (6.2)	5 (7.4)	1.19 (0.36-3.91)	.77	0.36-3.91	.77
Eyes do not converge	66 (58.9)	46 (67.6)	1.46 (0.77-2.74)	.24	0.71-3.00	.49
Pupillary diameter, median (IQR), mm^j^	5.5 (4.5-6.0)	5.5 (4.5-6.5)	1.11 (0.85-1.45)	.45	NA	NA
Total clues, median (IQR)	1.0 (0.0-1.0)	1.0 (0.0-1.0)	1.45 (0.80-2.60)	.22	0.78-2.68	.28
Modified Romberg Balance Test						
Instructions	30 (27.0)^g^	24 (35.3)	1.47 (0.77-2.82)	.24	0.66-3.26	.74
Internal clock, median (IQR), s^k^	33 (33.0-39.0)	32 (28.8-23.2)	0.98 (0.96-1.01)	.20	NA	NA
Internal clock (not acceptable)	51 (45.9)^g^	31 (45.6)	0.99 (0.54-1.81)	.96	0.54-1.81	.96
Eyelid tremors	91 (81.2)	52 (76.5)	0.75 (0.36-1.56)	.44	0.33-1.69	.74
Body tremors	29 (25.9)	19 (27.9)	1.11 (0.56-2.19)	.76	0.55-2.26	.95
Sways	75 (67.6)^g^	49 (73.1)^d^	1.31 (0.67-2.56)	.43	0.62-2.75	.74
Total clues, median (IQR)	2.0 (2.0-3.0)	2.0 (2.0-3.0)	1.13 (0.85-1.51)	.39	0.85-1.51	.39
Total clues for all tests, median (IQR)^d^^,^^e^	9.0 (7.0-11.2)	12.0 (9.0-14.0)	1.23 (1.11-1.36)	<.001	1.11-1.36	<.001

Abbreviations: FST, field sobriety test; NA, not applicable; OLS, One Leg Stand; SFST, standardized field sobriety test; THC, Δ^9^-tetrahydrocannabinol; WAT, Walk and Turn.

^a^ Data are presented as the number (percentage) of participants unless otherwise indicated.

^b^ Based on logistic regression assessing the associations with impairment.

^c^ Adjusted values were calculated using the false discovery rate method.

^d^ n = 67.

^e^ Estimates within treatments showed an association in the THC group only.

^f^ n = 111.

^g^ n = 110.

^h^ n = 66.

^i^ n = 65.

^j^ Numeric variable (not included in calculation of total clues).

^k^ Numeric variable for estimate of when 30 seconds had passed (not included in calculation of total clues).

## Discussion

In this randomized clinical trial, highly trained law enforcement officers found significantly worse FST performance in the THC group compared with the placebo group and correctly identified a greater proportion of the THC group as being exposed to THC. However, a substantial proportion of the placebo group performed poorly on the FSTs, and officers classified 49.2% of the placebo group as FST impaired. Of all participants who officers believed to have received THC whether they received THC or placebo, 92.8% were classified as FST impaired. The simulator-impaired group did worse on several FSTs, supporting the external validity of the tests.

### Individual FSTs

Field sobriety tests have shown mixed sensitivity to cannabis exposure and related impairments.^14,15,16,17,18,19^ In this study, 2 standardized FST measures (WAT and OLS) differentiated between the THC and placebo groups using alcohol-validated cut points; FTN and LOC clues also significantly differed. The MROM test was not sensitive to THC exposure.

Although cannabis is cited as affecting cognition more than motor skills, we found sway and balance to be sensitive to use. We found no differences in pupil sizes, suggesting that increased pupil size is not a universal indicator of exposure.^16,29^ Because participants who received THC estimated time more accurately than the placebo group, our data do not support this as an indicator of exposure.^30^

### Placebo Group FST Impairment

The prevalence of FST impairment in the placebo group (49.2%) is concerning since it is assumed that officers will adjust for confounding factors (eg, an injury). False-positive results on FSTs have been noted by other researchers.^13,31^ Newmeyer et al^16^ found that 35% of the placebo group had 2 or more clues on WAT or OLS, while Bosker et al^15^ reported an overall false-positive rate of 16% (58% on WAT, 21% on OLS). Lower rates have also been reported (eg, 0%-8.3%,^19^ 0%,^18^ and 2.5%-7.5%^21^). These studies did not use trained officers for FST administration.

The reasons for the placebo group doing poorly are not clear. There were no differences in demographics, THC use history, or treatment guess between FST-impaired and unimpaired participants. There were also no statistically significant differences in THC concentrations, which were at very low levels and unlikely to be functionally significant, or composite drive scores. The variability in composite drive scores is consistent with FSTs correlating with driving performance, and there was substantial overlap in composite drive score distributions. No specific clues were associated with being FST impaired.

It could be hypothesized that the FST-impaired participants showed residual effects from prior THC use, although studies on this topic are mixed.^32,33^ Also, we found no differences in use intensity or time since use and no evidence of residual effects on pretreatment simulator performance.^34^ The lack of large, blinded studies using officer-administered FSTs with nonintoxicated individuals is a substantial limitation in understanding the prevalence of poor FST performance in typical drivers.

### Officer Estimation of Treatment

Importantly, officer estimation regarding THC exposure was based on interactions during the FSTs, not a 12-step DRE evaluation (advanced approach to identifying impairing drugs).^35^ However, there are 2 findings of note. Of participants classified as FST impaired, officers strongly or somewhat believed that 99.2% of participants had received THC, suggesting they suspected all poorly performing participants to be under the influence. Conversely, almost all individuals (92.7%) who officers believed to have received THC were identified as FST impaired, in contrast to simulator results for these participants^5^ and many studies that have shown some overlap in performance of THC and placebo groups.^33,36,37^ The reason for the discrepancy between the omnipresence of FST impairment compared with simulator impairment is not clear. Since there is no impairment gold standard, one cannot assume that either indicates true impairment.

It is possible that physiological THC effects, including those seen on FSTs, were assumed by officers to indicate THC-associated impairment. The FSTs were validated using blood alcohol concentrations, which correlate with driving impairment and not with driving performance per se. Such relationships between THC concentrations and impairment do not hold with cannabis, and there are no validated physiological indicators of THC-related impairment. It is possible that under some conditions, officers may infer that indicators of recent THC exposure are causal with respect to FST impairments. (The effects of adding toxicology testing with FST results is addressed by Fitzgerald et al.^38^)

### FST Impairment Changes

Field sobriety test impairment declined in both groups over time. One might assume that the placebo group improvement reflects practice effects (and possibly officer expectations), whereas THC group improvement reflects recovery from THC and possible practice effects. Since the 2 groups had similar slopes, these cannot be differentiated with the binary FST impairment outcome. When using the better-dispersed total clues (which while not explicitly used by law enforcement for determining impairment, provide a possible indicator of FST changes), improvement in the THC group was greater than would be expected by practice effects alone, suggesting that the FSTs partially capture recovery from THC. The reduction in placebo group FST impairment rates suggests that FST exposures prior to treatment may have improved performance in previous studies,^15,18^ perhaps providing underestimates of the FST impairment rates that might be seen in nonintoxicated drivers at roadside.

### FSTs and Driving Simulator Performance

Previous controlled THC studies showed poor relationships between driving and FSTs, including impairments on the FSTs (61%-100%) but not on a simulator (which did not detect THC effects),^14^ and SFSTs (4.5-5.0 hours after oral THC ingestion) not detecting on-road driving impairments 2 to 4 hours after oral ingestion.^19^ The current study provides some validation that the 2 SFSTs (WAT and OLS) and other FSTs are generally sensitive to poor driving simulator performance, with participants impaired on the simulator having greater odds of poor FST performance compared with participants not impaired on the simulator (WAT: odds ratios from 2.00 [95% CI, 1.00-4.00] to 3.03 [95% CI, 1.62-5.68]). Components of WAT, OLS, and FTN and total clues were uniquely associated with poor driving in the THC group.

### Research Design, Expectancy, and Confirmation Bias

Aspects of the current study’s design may have influenced officer conclusions. Officers knew that participants were prescreened to exclude impairing substances and medical conditions and that a subset would receive THC. They may have been predisposed to anticipate (1) poor FST performance and (2) that poor performance would likely be due to THC exposure. This may have inflated FST sensitivity and possibly impacted secondary outcomes. Since 1 officer evaluated a single participant at all time points, he or she may have also expected recovery over time.

Officers also knew that many participants would receive placebo, and it is surprising that THC exposure was assumed in almost all individuals who performed poorly on the FSTs. Officers may encounter situations in which they suspect recent cannabis use (eg, noticing cannabis paraphernalia or odors, drivers stating that they use cannabis); such information could potentially influence the belief that poor FST performance may be causally related to cannabis use.

Cognitive,^39^ or confirmation, bias refers to seeking or interpreting evidence that supports existing beliefs or hypotheses often outside of awareness.^40^ Law enforcement in state-legal jurisdictions emphasizes that THC-related impairment, and not just exposure, is the question of interest. Confirmation bias, common in the general population, can remain despite advanced training (including in law enforcement and forensic sciences^23,41,42^), and it may have been a factor in this study.

This risk of bias reinforces the importance of the current practice of officers not knowing toxicology results prior to determining impairment status. It is unclear whether point-of-collection toxicology testing (eg, via oral fluid) may impact this practice.

### Detection of Impairment

Developing brief, objective measures that detect impairment yet show robust specificity (healthy adults performing without difficulty) is challenging. Even in controlled settings, cognitive testing is limited in identifying impaired drivers.^43^

Deducing whether a specific individual is impaired is also difficult.^44^ While the THC group in this study demonstrated FST deficits, the specificity for a single individual was poor. Officer training^10^ emphasizes that impairment is based on the totality of the evidence, including driving behavior and driver interviews. This study suggests that this is perhaps even more critical when attributing causality (eg, cannabis). A DRE evaluation may prove to be more informative,^35^ and well-controlled studies are needed.

The lack of a gold standard for driving impairment presents an ongoing challenge in the field. Although the outcome of interest is impairment in real-world driving situations, all experimentally controlled research uses surrogates—driving simulators, cognitive testing, FSTs, biofluid assays, or low-risk on-road evaluations (eg, no crash-avoidance challenges).

### Limitations

This study has limitations. Officers evaluated individuals based on select FSTs (not horizontal gaze nystagmus) and brief interactions without interview data or witnessing driving performance. Lacking a 12-step DRE evaluation, officers did not make a final determination that a participant was likely under the influence of cannabis; they made their best estimation based on FSTs and study interactions. The FSTs were administered at 70 minutes. Peak drug effects may take place earlier,^45,46,47^ although previous analyses of the simulator performance in this cohort showed that simulator recovery did not occur until 90 minutes to 3 hours 30 minutes after smoking cannabis,^5^ and 70 minutes from smoking to FST initiation remains relevant to a period that might be encountered in a roadside scenario. We only examined smoked cannabis; other products (eg, high-THC concentrates) may have different FST effects. Although some have expressed concerns regarding the low THC content of National Institute on Drug Abuse cannabis,^48^ participants reported a median highness level of 64 on a scale of 0 to 100, suggesting the content was sufficient to achieve significant intoxication. The impact of higher-THC flower products is unclear, although users often self-titrate and may not necessarily become more impaired by such products.^5^

## Conclusions

This randomized clinical trial found that FSTs administered by highly trained law enforcement officers differentiated between individuals receiving THC vs placebo and that driving abilities were associated with results of some FSTs. However, participants receiving placebo had a high rate of inadequate performance of FSTs, and officers frequently suspected poor FST performance to be due to THC-related impairment. Road safety is a critical issue in an era of increasing cannabis legalization. This requires a comprehensive effort, including public information and prevention efforts to keep impaired drivers off the road. Once they are on the road, law enforcement plays a critical role in removing impaired drivers. The findings of this study suggest that (1) FSTs are useful adjuncts but do not provide strong objective evidence of THC-specific impairment and (2) additional efforts to validate existing methods and provide law enforcement with new, effective tools for identifying impairment are needed.

## Notes

Supplement 1.
Trial Protocol

Supplement 2. **eTable 1.** Field Sobriety Tests Used in the Current Study **eTable 2.** Percentage of Participants Classified as FST-Impaired, by Officer Estimation Regarding Treatment Assignment **eTable 3.** Percentage of Participants Classified as FST-Impaired, by What the Officer Believed Was the Person’s Treatment Assignment **eTable 4.** Percentage of Participants Showing an FST Clue at Each Time Point by Treatment Arm Among Those Classified as FST-Impaired at the First FST Evaluation **eTable 5.** Association Between Demographic Characteristics and Officer-Determined FST Impairment at 71 Minutes After Smoking in the Placebo Group **eTable 6.** Univariable Associations Between FST Items and Being Classified as FST-Impaired by the Officer in the Placebo Group **eAppendix.** Driving Simulations, Randomization and Blinding, Power and Sample Size, and Adverse Events
eReferences

Supplement 3.
Data Sharing Statement
