# Supplementary material for: Evaluation of Field Sobriety Tests for Identifying Drivers Under the Influence of Cannabis: A Randomized Clinical Trial
Source: JAMA Psychiatry. 2023 Aug 2;80(9):914–23. doi: 10.1001/jamapsychiatry.2023.2345 (PMC10398547; doi:10.1001/jamapsychiatry.2023.2345)
Supplement: Supplement 1. — Trial Protocol [file jamapsychiatry-e232345-s001.pdf]

A Randomized, Controlled Trial of Cannabis in Healthy Volunteers Evaluating Simulated Driving, Field Performance Tests and Cannabinoid Levels

RESEARCH PROTOCOL
May 21, 2020

TABLE OF CONTENTS

PROJECT TITLE 2
PRINCIPAL INVESTIGATOR 2
CO-INVESTIGATORS 2
FACILITIES 2
ESTIMATED DURATION OF THE STUDY 2
LAY LANGUAGE SUMMARY OR SYNOPSIS 2
SPECIFIC AIMS 2
BACKGROUND AND SIGNIFICANCE 3
RESEARCH DESIGN AND METHODS 4
  Participants 4
  Visits 4
  Supply and Administration of Cannabis 5
  Assessments during Experimental Visits 6
  Drug Recognition Expert Field Sobriety Test 7
  Performance-based tablet assessments 7
  Success of Blinding 8
  Psychoactive Side Effects During Driving Simulation Sessions and After Consuming the Study Drug 8
  Assays for Δ9-THC in Blood 8
  Identification of Recent Cannabis Intake Using Whole Blood 9
  Assays for Oral Fluid 9
  Assays for Breath Specimens 10
  Statistical Analysis Plan 10
HUMAN SUBJECTS 11
  Inclusion criteria 12
  Exclusion criteria 12
  Recruitment and Procedures Preparatory to Research 12
  Informed Consent 13
  Potential Risks 14
  Risk Management Procedures and Adequacy of Resources 16
  Data and Safety Monitoring Board (DSMB) 16
  Privacy and Confidentiality Considerations Including Data Access and Management 17
  Potential Benefits 17
  Risk/Benefit Ratio 18
  Expense to Participant 18
  Compensation for Participation 18
FUNDING SUPPORT 18
DRUG PROCESSING 18
BIBLIOGRAPHY 19

PROJECT TITLE

A Randomized, Controlled Trial of Cannabis in Healthy Volunteers Evaluating Simulated Driving, Field Performance Tests and Cannabinoid Levels

PRINCIPAL INVESTIGATOR

Thomas D. Marcotte PhD, Professor, Department of Psychiatry

CO-INVESTIGATORS

Robert L. Fitzgerald, PhD, Professor, Department of Pathology
David J. Grelotti, MD, Associate Professor, Department of Psychiatry

FACILITIES

The study will be conducted at the Center for Medicinal Cannabis Research (CMCR), which is co-located with the HIV Neurobehavioral Research Program (HNRP) facility at 220 Dickinson Street, MC8231, San Diego, CA 92103-8231. Two rooms have been outfitted with a negative pressure system to enable cannabis to be vented to the atmosphere without contaminating the workspace of others working in this building. We will use the Investigational Drug Service 200 West Arbor Drive, Suite 1-317, San Diego, CA 92103-8765, for the storage of cannabis.

ESTIMATED DURATION OF THE STUDY

It is estimated that the study will take 3 years.

LAY LANGUAGE SUMMARY OR SYNOPSIS

This study was authorized by the California Legislature (Assembly Bill 266, the Medical Marijuana Regulation and Safety Act)[1] to help with detection of driving under the influence of cannabis. Healthy volunteers will inhale smoked cannabis with either 0.02% (placebo), 5.9%, or 13.4% Δ9-tetrahydrocannbinol (THC) at the beginning of the day, and complete driving simulations, iPad-based performance assessments, and bodily fluid draws (e.g., blood, oral fluid [OF], breath) before the cannabis smoking and over the subsequent 6 hours. The first specific aims address the relationship of the dose of Δ9-THC on driving performance and the duration of driving impairment in terms of hours from initial use.

SPECIFIC AIMS

The first specific Aims are:

Aim 1 To determine the impact of Δ9-THC dose on driving performance.

> *Hypothesis 1.* During an 8-hour driving simulation session, 0.02% (placebo), 5.9%, and 13.4% Δ9-THC will demonstrate a stair-step progression in worsening on the Composite Drive Score, a composite of key driving variables. Participants' driving performance will be worse under the influence of 13.4% Δ9-THC than under the influence of 5.9% Δ9-THC, which in turn will be worse than when a participant is under the influence of placebo Δ9-THC.

Aim 2 To determine the time course of driving impairment in terms of hours from initial use.

> *Hypothesis 2.* During the six hours post inhalation of cannabis, 13.4%, 5.9%, and 0.02% (placebo) Δ9-THC will demonstrate a stair-step pattern with respect to the recovery from the effects of cannabis on driving performance. By this is meant that reduced driving performance under the influence of 13.4% Δ9-THC will last longer than 5.9% Δ9-THC, which in turn will last longer than when a participant is under the influence of placebo Δ9-THC.

BACKGROUND AND SIGNIFICANCE

There are several studies suggesting that higher doses of whole-blood or plasma Δ9-THC concentration are associated with increased crash risk and crash culpability[2-4]. However, attempts to define a cut-off point for blood Δ9-THC levels have proven to be challenging. Unlike alcohol, for which a level can be reasonably measured using a breathalyzer (and confirmed with a blood test), detection of a cut-off point for intoxication related to Δ9-THC concentration has eluded scientific verification. Recent evidence suggests blood Δ9-THC concentrations of 2-5 ng/mL are associated with substantial driving impairment, particularly in occasional smokers[5]. Others have countered that this level leads to false positives, particularly in heavy cannabis users inasmuch as THC may be detectable in their blood specimens for 12-24 hours after inhalation[6]. Given that 12 to 24 hours is beyond the likely period of driving impairment[7], this would appear to be a justifiable objection to a per se cut-off point for a Δ9-THC concentration indicative of impairment. Maximal driving impairment is found 20 to 40 minutes after smoking, and the risk of driving impairment may decrease after 2.5 hours, at least in those who smoke 18 mg Δ9-THC or less, the dose often used experimentally to duplicate a single joint[7]. Other studies, however, report residual MVA crash risk when cannabis is used within 4 hours prior to driving[2,3,8-10].

The roadside examination using the Standardized Field Sobriety Test (SFST) for proof of cannabis-related impairment has not been an ideal alternative to blood levels. Originally devised to evaluate impairment under the influence of alcohol, the SFST is comprised of three examinations administered in a standardized manner by law enforcement officers. The 'Horizontal Gaze Nystagmus' (HGN), the 'One Leg Stand' (OLS) and the 'Walk and Turn' test (WAT) require a person to follow instructions and perform motor activities. During the assessments, officers observe and record signs of impairment. In one study, Δ9-THC produced impairments on overall SFST performance in only 50 % of the participants[11]. In a separate study involving acute administration of cannabis, 30% of people failed the SFST[12]. This discrepancy in rate of failure was thought to be in part due to the participant's cannabis use history. The reported frequency of cannabis use varied from once a week to once every 2–6 months in the study where there was the SFSTs identified impairment in 50% of the participants. The other study included more frequent users who smoked cannabis on at least four occasions per week. Previous studies demonstrated that heavy cannabis users develop tolerance to the impairing effects of Δ9-THC on neurocognitive measures[13,14]. The same phenomena may hold true for the SFSTs.

Based upon the above, another means to help law enforcement officers discern driving under the influence of cannabis would be helpful. One future possibility is the development of performance-based measures of cannabis-related impairments. We have developed brief tablet-based measures in order to be practicably administered repeatedly over a short time period, that if successful in the future could be used in the field by law enforcement officers (e.g., a cannabis-focused field sobriety test).

Although blood and plasma levels leave a lot to be desired in terms of a cut-off point for impaired driving, there is still a great deal of interest in biological markers among law makers. In all probability, oral fluid will probably become the most prevalent matrix for roadside screening[15]. The rationale is that legislators and police officers will desire rapid analysis of driving under the influence of cannabis testing at the roadside, eliminating transport of detainees to hospitals or police stations for a phlebotomy to determine a blood level. Moreover, knowing the time cannabis was last used is important for determining impairment in crash investigations. Currently, this is performed using whole blood or plasma. Consequently, a correlation for the time estimate of marijuana exposure from a more contemporaneous matrix (e.g., oral fluid) would be of value. Two decades ago, two models for predicting time of last cannabis use from single plasma cannabinoid concentrations were devised. Model I used simple regression with the 9-tetrahydrocannabinol (THC) concentration, while model II used a simple regression equation with the ratio of 11-nor-9-carboxy-THC (THCCOOH) to THC[16]. An objective of the current study will be to extend such studies using analyses of cannabinoids in saliva and breath.

Detection of Δ9-THC in oral fluid (OF) has been associated with a strong contamination of the oral cavity during smoking and to a recent cannabis use. Δ9-THC and its metabolites are poorly excreted from the blood and tissues into this matrix. In line with these observations, analysis of OF revealed very high concentrations of THC in OF just after cannabis smoking, while 11- hydroxy-THC (11-OH-THC) was not detected and only trace amounts of THCCOOH were found when measured[17]. Studies that used intravenous administration of THC

have suggested that the transfer of THC from the blood into OF is limited[18]. Since THCCOOH is not known to be present in cannabis smoke, its detection in OF could only result from active cannabis consumption.

Breath may offer an alternative matrix for testing for recent driving under the influence of cannabis, but is limited to a short detection window (0.5–2 h)[19]. In the present study, we will use the SensAbues breath collection device and a validated liquid chromatography–tandem mass spectrometry (LC-MS/MS) method to quantify breath cannabinoids in study participants following controlled smoked THC administration. The time of last cannabis use from the breath Δ9-THC concentration will be correlated with that from blood levels of this cannabinoid over 2 hours after cannabis intake. In a previous study, no breath sample tested positive for THCCOOH[19].

RESEARCH DESIGN AND METHODS

Participants

Two hundred and forty healthy individuals will be recruited with the intention to study 180 participants who meet inclusion/exclusion criteria and ultimately provide complete data. Participants will have used cannabis at least four times in the preceding month, as determined by self-report. They will be randomized to receive either 0.02% (placebo; n = 60), 5.9% (n = 60), or 13.4% (n = 60) Δ9-THC.

Visits

Participants will complete a screening interview and baseline visit, and an experimental visit on separate days as part of this study to include the following:

- Screening + Baseline visit (Day 1 – a 4-hour visit). A review of medical and substance use history, as well as a safety evaluation (including relevant labs, if indicated by findings on history or physical exam). In addition, participants will complete a series of driving simulations in order to orient them to the simulator, establish baseline levels of performance and minimize the effects associated with repeated exposure to the simulations (practice effects). Participants will also be assessed for far visual acuity (ETDRS eye chart), color vision (Ishirara), and contrast sensitivity (Pelli-Robson Chart).

- Experimental visit. After completing a pre-smoking driving simulation and iPad testing, participants will inhale smoked cannabis with either 0.02% (placebo), 5.9%, or 13.4% Δ9-THC beginning of the day. They will then complete driving simulator assessments, iPad-based performance assessments, and fluid draws (blood, saliva, breath) as indicated in Table 1 below. By conducting repeated assessments following intake of the study drug, we will be able to determine at what point participants no longer exhibit acute effects for each of the drugs. The acute effects will be measurable using driving simulation, field sobriety tests, and neuropsychological testing via an iPad.

Subjects will undergo a urine drug screen and breathalyzer for alcohol and drugs at the beginning of the screening/baseline visit and the experimental visit. In addition, an oral fluid sample will be run for the presence of delta-9 THC using a testing device (Draeger 5000, Houston, TX) employed by some law enforcement officers to detect recent cannabis use. An oral fluid value of > 5ng suggests recent use. Thus, should the oral fluid sample indicate > 5ng THC, the assessment may be canceled and rescheduled, since participants are to have abstained from use for at least 2 days. Additional samples may be sent for confirmatory testing if the results of the urine drug screen or Draeger are inconsistent with participant report. (Note that since there is no practical way to confirm non-use in recent days [short of an inpatient setting], we will also be collecting blood samples prior to intake of study cannabis and perform a confirmatory analysis with mass spectroscopy/gas chromatography. This may later be considered in our analyses of study findings.)

Table 1. General Outline Assessment of Study Timeline (in minutes)

	Pre		0	30	60	90	120	170	210	255	280	320
Vitals	X	Smoke Cannabis Cigarettes	X			X		X	X			X
OF Collection	X		X			X			X		X	
Breath Collection	X		X			X			X		X	
Blood Draw	X		X		X	X	X	X	X	X	X	X
Driving Simulations	X			X		X			X		X	
SFSTs	*				X			X	X		X	
iPad test	X				X		X			X		X

Supply and Administration of Cannabis

Cannabis will be harvested at the University of Mississippi under the supervision of the National Institute on Drug Abuse (NIDA). Our IND application for cannabis as an obligatory part of federal regulations for obtaining NIDA cannabis has been approved by the FDA (for details, please see below in Section 11 under Procedures Preparatory To Research). NIDA will be able to provide bulk cannabis for this study in the concentrations mentioned above. Bulk placebo is made from whole plant with extraction of cannabinoids and has the natural smell and appearance of the active cannabis. After overnight delivery, the bulk cannabis will be stored in a freezer securely bolted to the floor of the UCSD Research Pharmacy at Hillcrest. Further precautions against theft of the study drug included limited password access to the pharmacy, with a state-of-the-art entry detection system and a direct connection of the alarm system of the room housing the freezer to the Hospital Police Department.

After informed consent is obtained and eligibility determined participants will be scheduled for a baseline session and one, 8-hour experimental session at the CMCR (220 Dickinson St, San Diego, CA 92103). Participants will receive 5.9%, 13.4%, or placebo (0.02% THC) cannabis cigarettes at their visit. Group assignment will be assigned using a permuted blocks randomization with stratification by prior cannabis exposure (frequent user [≥4x per week] versus occasional user [≤4x per week]). The allocation schedule will be kept in the pharmacy and concealed from other study personnel. Patients and assessors will be blinded to group assignments. The cigarettes will be stored in a freezer at -20°C until the day before use. At least 1 hour before the study session, enough bulk cannabis (0.7 g) to roll one marijuana cigarette will be thawed. The cannabis cigarette will be hand-rolled by a licensed clinician before the study session. The adhesive seal on the rolling paper will be activated with drops of sterile water.

An ad libitum cannabis smoking will be utilized, with a maximum smoking time of 10 min or until the cigarette is smoked until the participant cannot hold it longer using clips. This will likely insure that enough THC is consumed to allow OF concentrations of this cannabinoid to be accumulated while, at the same time, protecting the participant from their finger being burned from the proximal end of the cigarette. In practice, the participant will be instructed to "*Smoke the cigarette the way you do at home to get high. You may take up to 10 minutes.*"

However, it will not be mandatory for participants to inhale enough cannabis to incinerate the cigarette until they can no longer hold it. As an alternative, the participant may signal by raising their hand that they are not tolerant of further dosing for whatever reason. It has been stated, "An experienced cannabis smoker can titrate and regulate dose to obtain the desired acute effects and to minimize undesired effects"[20]. Though not mandatory to incinerate the cigarette to the proximal tip, a minimum of 4 puffs will be required for a participant to remain in the study. Otherwise, we run the risk of having someone undergoing assessments without being under a minimum amount of intoxication from cannabis.

A nurse will continuously supervise the participant during the smoking session through a viewing window in an adjoining room with an intercom and insure that they are progressing safely. A physician will be readily available for consultation.

Assessments during Experimental Visits

Driving simulations: Simulation hardware consist of a 3-screen, wide field-of-view monitor setup, steering wheel, and accelerator and brake pedals (Figure 1). The fully interactive simulations will assess lane tracking (standard deviation of lateral position [SDLP], or "weaving"), response to divided attention stimuli (accuracy, response time), car following, and performance during scenarios simulating routine driving as well as crash avoidance situations.

Participants in the simulator study will also be assessed for far visual acuity (Snellen Visual Acuity eye chart), color vision, and contrast sensitivity (Vistech Contrast Sensitivity (Pelli-Robson Chart)). Participants will complete an orientation and practice drives prior to the initial simulation, in order to familiarize them with the controls and roadways.

- <u>Lane Tracking/Divided attention:</u> Participants will be instructed to maintain their lane position and speed, and respond to divided attention stimuli on an adjacent iPad. The primary outcomes are standard deviation of lateral deviation (SDLP), latency and accuracy on the divided attention tasks, and speed deviation. SDLP is a measure of how well subjects maintain their lane position, providing an index for each subject's road tracking error and ability to control the lateral motion of the car. It is primarily controlled by automatic information processing and outside of conscious control. SDLP has been shown to be sensitive to the effects of drugs in both on-road and simulator studies[21-25]. It has been examined in individuals under the influence of alcohol, marijuana, and MDMA, as well as with neurologic populations[26-30]. SDLP has also demonstrated good test-retest reliability over short and long-term follow-ups[31-34].
- The <u>Divided Attention task</u> will be a modification of the Surrogate Reference Task (SuRT)[35]. The primary outcomes on this component are response latency and accuracy on the mSuRT tasks. The mSuRT is a visual perceptual task which presents subjects with an approximately 8" touch screen filled with circles and requires participants to point to a target circle (Figure 1). The level of difficulty is varied by changing the ratio of the size of the distractor circles and target circles. The equipment will be to the side of the monitor. The SuRT is a measure of performance under high cognitive load and controlled processing, in that participants must divide their attention among three stimuli (roadway, speedometer, and events in the periphery), and is reflective of the workload generated by a real task (e.g., a GPS system). Face valid tasks such as navigation destination entry draw attention away from the road in highly variable ways (i.e. there tend to be large differences in how people attack problems associated with complex interactions). On the other hand, surrogate or structured tasks allow us to look at changes in attention in a more controlled fashion. This will enable us to address how participants under the influence of cannabis vary allocation strategies with workload.

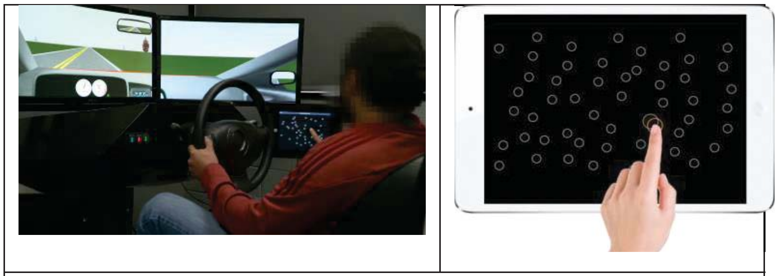

Figure 1. Simulator and Modified Surrogate Reference Task (mSuRT)

- <u>Car Following</u>: The primary outcomes are (1) coherence between the participant and lead cars (a general correlation [0–1] of the participant's ability to accurately track the speed variations of the lead car); (2) time delay (or the reaction time to changes in the lead car's speed); and 3) distance from the lead car. The subject is to adjust his/her speed to a lead car that speeds up and slows down according to a sinusoidal wave.

- Crash avoidance/decision-making: In order to assess treatment effects during routine and non-routine events we will include scenarios addressing 1) the “yellow light dilemma”, wherein individuals need to respond to a yellow light onset by abruptly braking (risking a rear-end collision), or go through the intersection (risking running a red light), and 2) crash avoidance. Participants will be instructed to drive 45mph, and will encounter 8 green traffic lights, 4 of which will switch to yellow. These will be randomized within each drive. Consistent with California law[36], the yellow light phase (time before the yellow light turns red) will be 4.3s. The time available to perceive and respond to the yellow light will be held constant for all participants by controlling initiation of the yellow light by using the vehicle’s velocity to determine the time-to-location (start of intersection). This will be set at 3.4, 3.0, 2.7 and 2.2s, settings which in previous studies have shown to elicit a range of responses (running the yellow light, stopping) [37,38]. The primary outcomes will be stop/go percent and perception-reaction time (PRT; time of yellow onset to start braking or accelerating through the intersection), although a number of additional behavioral outcomes will be of interest. The simulation will also include a crash avoidance scenario in which the participant drives down a visually complex roadway (moving cars, pedestrians) and encounters the sudden appearance of a pedestrian, or car pulling out, in the roadway. Primary outcomes are the PRT to the incursion, and whether a collision occurs. Since an important aspect of this task is the unexpected nature of the event, the incursion point and object (vehicle, pedestrian) will vary across assessments (but be consistent across all participants).

Additional components of the simulation will include left hand turns across traffic (assessing gap acceptance), freeway off ramps, merging into traffic, and brief audio driving instructions (requiring intact short-term memory).

Overall driving performance will be the Composite Drive Score. The Composite Drive Score incorporates the key variables from the more controlled scenarios above (Lane Tracking/Divided Attention and Car Following) and combine them in a manner to create a single score. We will create a baseline anchor for performance based upon the performance of all participants during their pre-smoking drive. Subsequent Composite Drive Scores will use this as the basis for developing the change score (from pre-smoking) at each timepoint.

In order to accomplish this, z-scores will established based upon the pre-smoking simulator performance, using the mean and standard deviation on each score for all participants. Z-scores for each participant will be calculated by subtracting the group mean score from the participant’s score and dividing that by the group standard deviation (so that, in the end, at the pre-smoke driving the Composite Drive Score for the entire sample will have a mean z-score of 0, with a standard deviation of 1). Higher z-scores at each timepoint will indicate worse performance. When examining the change in Composite Drive Score, a higher score will indicate a decline in performance (e.g., Time 2 minus Time 1). The Composite Drive Score will be comprised of: mSuRT task (SDLP, Speed Deviation, correct hits on SuRT) and Car Following (coherence).

Drug Recognition Expert Field Sobriety Test

Several psychophysical tests from among those used by Drug Recognition Experts (DRE) for assessment of driving under the influence of cannabis will be performed.

- Modified Romberg test (mROM)
- Lack of Convergence (LOC)
- Finger-to-Nose (FTN)
- Walk and Turn (WAT)
- One Leg Stand (OLS)

To insure proper administration, field sobriety tests will be performed by DRE instructors.

Performance-based tablet assessments

The following will be performed using an iPad with software designed by Digital Artefacts LLC (Iowa City, IA) based upon collaboratively-established specifications. The iPad assessments will take approximately 10 minutes:

- Lane Tracking. This test assesses the participant's ability to adapt to an error signal in a first-order compensatory task, and has been shown to be sensitive to the effects of Δ9-THC[14,39-41]. This will be assessed by the participant keeping a solid circle within two boundary lines by swiveling the iPad. The participant must overcome built-in error in horizontal deviation.
- Dual Attention. The participant will follow a moving target (square) with a stylus, with a secondary square located elsewhere on the screen. The secondary stimulus will change colors and shapes. When the secondary square turns a specific color, the participant is to switch over to tracking that stimulus.
- Time estimation. Cannabis can affect time perception and estimation. Deficits in temporal processing could have significant implications for driving, for example in estimating the amount of time available to pass through a yellow light, or anticipating cross-traffic. We will thus administer a brief measure of time estimation. As recommended by Sewell et al.[42], we will use an approach that minimizes the use of subvocal counting, which may artificially decrease variation that might occur during real-world multi-tasking. Five trials, with randomly generated durations ranging from 5 to 30s (e.g., 7, 11, 29, 14, 23 seconds), will be generated. During each assessment, participants will be presented with the letter M in random parts of the iPad screen. The participant is then to count the number of "M"s that appear on the screen, at which point he/she is to select the number of "M"s and the amount of time that has elapsed. The primary outcome is the ratio of estimated time to actual time.
- Balance. This has proven to one of the more sensitive, but challenging, aspects to the DRE Field Tests since sway is subjectively determined by the officer. During the modified Romberg Test, forward-backward, and lateral postural sway will be assessed via the accelerometer and gyroscope features of a TI Simple Link Sensor Tag strapped to the back of the participant, using a Velcro belt.
- Visual Spatial Memory Learning Test. Cannabis can affect memory acutely[13,43,44]. We will assess short term memory using a visual-spatial learning test (VSLT). This test is modeled after other tests of visuospatial memory (e.g., the Brief Visuospatial Memory Test-Revised, Visual Spatial Learning Test). The test requires the subject to a) memorize 4 designs that are difficult to verbally encode, b) recognize them among a group of 8 designs (5 foils) and c) recall the correct placement of these designs on a 3 X 3 matrix. Participants will complete three trials. Since there is evidence that cannabis effects are more pronounced the longer the delay between presentation and recall[45], the delay between stimulus presentation and time to recall will be 4, 12, and 24 seconds for the successive trials. The score is the number of figures correctly identified and placed.

Success of Blinding

Twice during the experimental session, we will ask questions about successful blinding of the study drug. The method will be to ask participants ''Which treatment do you think you received (or were assigned to)?''

- ☐ I strongly believe I received real marijuana
- ☐ I somewhat believe I received real marijuana
- ☐ I do not know
- ☐ I somewhat believe I received placebo ("like a sugar pill")
- ☐ I strongly believe I received placebo ("like a sugar pill")

Psychoactive Side Effects During Driving Simulation Sessions and After Consuming the Study Drug

We will ask participants to evaluate their feeling side effects from cannabis (stoned, high, like the drug effect, feel impaired to drive) using VAS 0 to 100 scales with appropriate anchors.

Assays for Δ9-THC in Blood

In habitual, daily users, plasma Δ9-THC concentrations range from 1.0 to 11.0 ng/ml and are maintained by sequestration of the drug from the tissues[46]. This residual blood concentration makes setting thresholds for drug-driving legislation difficult because of the variability in the determination of concentration across individuals. Selecting a high cutoff will miss many impaired occasional users while selecting a low cutoff may pick up residual concentrations in frequent users. But there is clearly a case for defining such levels. Determining minimum blood, saliva and breath THC concentrations at which a driver becomes sufficiently

impaired to be unable to safely drive a vehicle is of particular concern given the increasing medicinal use of the drug. International legislation for driving under the influence of drugs (DUID) is based on either a proof of impairment or a per se approach. For the latter, this can be either zero-tolerance or based on concentration limits such as those used for alcohol.

During the driving simulator assessment, we will determine blood levels after administration of the study drug in order to evaluate the effects of Δ9-THC and on driving and cognition. An arm vein will be cannulated with an indwelling catheter. Blood will be collected in grey top (EDTA) vacutainer tubes The blood will be transferred to 1.8 ml cryovials and aliquots will be stored at –70C. Up to 108 cc or 21.6 teaspoons of blood will be collected.

Δ9-THC and metabolites will be quantified using isotope dilution ultra-performance liquid chromatography (UPLC) and tandem mass spectrometry (MS/MS) using methodologies similar to what have been published. Briefly, deuterium labeled internal standards will be added and proteins will be precipitated using acetonitrile. Δ9-THC and CBD will be isolated using solid phase extraction and analyzed using electrospray ionization. Δ9-THC will be analyzed using positive ion electrospray while negative ion ESI will be used for CBD using Waters Xevo TQS equipped with Waters Acquity UPLC. The limit of quantification (LOQ) will be 0.5 ng/mL of each of the components in whole blood. Our laboratory at UCSD has been using similar methodologies to accurately quantify small molecules for many years[47].

Identification of Recent Cannabis Intake Using Whole Blood

Human whole-blood cannabinoid data following cannabis smoking may assist in the identification of recent cannabis intake[48]. It has been hypothesized that several cannabinoids (e.g., THC-glucuronide, cannabidiol and cannabinol) might be useful for estimating the last time of cannabis intake. This follows from the finding that analytes of these cannabinoids, at observed Cmax, were not detected beyond 2 h after smoking, rendering them possible candidates for markers of recent cannabis smoking. However, they are not universally detectable in everyone after consuming cannabis; whole-blood (plasma) detection rates were as shown below in one study that involved occasional smokers (cannabis smoking at least twice monthly for 3 months before entry)[48].

Analyte	Whole blood	Plasma
THC-glucuronide	50%	80%
Cannabidiol	60%	80%
Cannabinol	80%	90%

These somewhat low observed detection rates render THC-glucuronide, CBD and CBN an inclusionary, but not exclusionary, marker for recent cannabis intake at a 0.5 ng/ml limit of quantification. Furthermore, CBD and CBN were not detectable after 1 h in either plasma or blood (limits of quantification [LOQ 1.0 ng/ml]). CBD and CBN had similar detection windows in whole blood and plasma, with CBN more prevalent than CBD between 0.25 and 1 h. CBD and CBN are amenable to GC-MS analysis, are often readily extracted by current mixed-mode solid-phase extraction procedures, and have commercially available deuterium-labeled internal standards, unlike cannabinoid glucuronides[48]. However, concentrations of these analytes in cannabis vary depending on chemovar[49] and storage time and conditions [50], potentially altering detection rates. Additionally, these cannabinoids are present in cannabis smoke[51,52] and, unlike THC-glucuronide, could possibly be detected in oral fluid. If detection limits improve for these minor cannabinoids (currently LOQ 1.0 ng/ml), further study could suggest potential cutoffs and analytical approaches for confirming these analytes as markers of recent cannabis intake. In the present study, we will analyze THC-glucuronide in whole blood as well as in oral fluid and breath to see if this cannabinoid would offer a recognizable marker of recent use of cannabis.

Assays for Oral Fluid

Oral fluid (OF) is becoming increasingly popular in many areas of drug testing as a diagnostic fluid, partly due to the ease and noninvasiveness of collection[53]. Oral fluid analysis for Δ9-THC and other drugs of abuse are

being reported in roadside testing[54,55].

OF samples will be collected a few minutes before inhalation (t =0 h) and afterwards. Specimens will be stored for a few hours at room temperature before refrigerating. We will employ the Quantisal™ collection device (Alere Inc., San Diego, CA) to collect and store saliva samples. Using ultra-performance liquid chromatography (UPLC) and tandem mass spectrometry (MS/MS), the levels of Δ9-THC in the saliva samples will be determined. We will base the OF LC/MS/MS assay on the method developed for blood analysis after optimizing extraction conditions for OF specimens. The OF Δ9-THC concentrations will be correlated with that from blood over 3.5 hours after cannabis intake.

Assays for Breath Specimens

Exhaled breath has recently been identified as a matrix for the detection of drugs of abuse including Δ9-THC [56]. This technology is based on a collecting device, the Drug Trap® (SensAbues AB, Sweden), with a filter which traps aerosols from breath. These aerosols mimic the blood in terms of the content of certain substances including Δ9-THC[56]. However, although exhaled breath may offer an alternative body material for identifying recent driving under the influence of cannabis, currently its sensitivity is limited to a short detection window (0.5–2 h)[19]. In that study, the number of individuals who had THC in their breath was reported. Among chronic smokers (n = 13), all breath samples were positive for THC at 0.89 h, 76.9% at 1.38 h, and 53.8% at 2.38 h, and only 1 sample was positive at 4.2 h after smoking. Among occasional smokers (n = 11), 90.9% of breath samples were THC-positive at 0.95 h and 63.6% at 1.49 h. One occasional smoker had no detectable THC.

Breath samples will be collected at baseline and then approximately 22, 99, 227 and 298 minutes post smoking with the SensAbues device (over a 3 min collection period).

SensAbues devices contain a mouthpiece and polymeric filter pad enclosed in a plastic collection chamber [56]. Devices protect against oral fluid contamination during sampling with barrier ledges inside the mouthpiece[56]. Participants will be asked to breathe normally, inhaling through their nose and exhaling through the SensAbues mouthpiece during sampling. After this is completed, the collection device is carefully opened and the filter removed with forceps, placed inside a small plastic bag and frozen at -70 degrees C. We will not leave the filter inside the SensAbues device and will insure that oral fluid does not get onto the filter. Food and beverage intake will be restricted 10 min before each collection. The collections will occur in a different room from the smoking room.

Using ultra-performance liquid chromatography (UPLC) and tandem mass spectrometry (MS/MS), breath concentrations of Δ9-THC will be determined using the basic LC/MS/MS procedures developed above after optimizing extraction conditions for measurement of THC from the OF collection device. The breath Δ9-THC concentrations will be correlated with that from blood levels of this cannabinoid over 2 hours after cannabis intake.

The following specimens will be obtained:

a. For blood target compounds will include (−)-trans-Δ9-tetrahydrocannabinol (THC), 11-hydroxy-Δ9-tetrahydrocannabinol (11-OH-THC), 11-nor-9-carboxy-delta-9-tetrahydrocannabinol (THC-COOH), Δ9-tetrahydrocannabinol-glucuronide (THC-glucuronide), cannabidiol (CBD), and cannabinol CBN. Blood specimens in grey top (naf) tubes
b. For oral fluid target compounds will include (−)-trans-Δ9-tetrahydrocannabinol (THC), cannabidiol (CBD), and cannabinol CBN.
c. For breath target compounds will include (−)-trans-Δ9-tetrahydrocannabinol (THC)

Statistical Analysis Plan

All tests will be two-sided and deemed significant if $p<0.05$, unless specified otherwise. Parallel design is assumed for the total sample N=180 with N=60 subject per group, where groups are defined as

control/placebo (0.02% Δ9-THC), 5.9% Δ9-THC, and 13.4% THC. Baseline demographic, medical, and psychiatric characteristics will be reported separately for each group as mean (standard deviation) or median (interquartile range) for numeric measures and as N (percent, %) for categorical measures. The baseline characteristics will be compared between groups using ANOVA for numeric variables and Chi-square test for categorical variables. Power transformations of skewed variables or non-parametric alternatives will be used, where appropriate. All assumptions will be checked prior to testing. Differences in baseline characteristics between the groups are not expected due to randomization. However, if they happen by chance, variables that differ between the groups will be considered as covariates in multivariable methods, as appropriate.

The primary analysis will focus on testing aims and initial hypotheses outlined above. The primary variables of interest will be measured at multiple time points with the goal of assessing how they change throughout the day, thus we will use statistical methods appropriate for analysis of data in repeated measures and longitudinal study designs.

Demographic and other relevant characteristics will be compared between groups using ANOVA, Kruskal-Wallis test, chi-square test, and Fisher's exact test as appropriate. Two-group comparisons will be carried out using t-test (or Wilcoxon), chi-square test, or Fisher's exact test. To meet the assumption of normality, some continuous variables may be standardized. Effect sizes for continuous outcomes will be estimated by Cohen's d or by Cliff's delta. Confidence intervals (CI) at 95% level will be calculated for all effect sizes. Confidence intervals reported with p-values adjusted for multiple testing will also be corrected using false discovery (FDR) method.

Generalized least squares models will be used for numeric outcomes with covariance structure selected by minimum Akaike Information Criterion (AIC). Poisson and logistic regression models with generalized estimating equation (GEE) method will be used for discrete and binary outcomes, respectively. Time will be treated as a factor to accommodate non-linear changes in the outcomes. Treatment will first be considered as a three-level variable - Placebo, 5.9% THC, and 13.4% THC. For all models, three terms will be included: treatment, time (5 time points), and treatment-time interaction. For effect sizes estimating differences at multiple time points, correction for multiple comparisons will be applied using false discovery rate (FDR) method (secondary analyses only).

Power. For power calculations, it was assumed that the placebo group will show minimal changes in Composite Drive Score (CDS) over time and that the 13.4% THC group will show a worsening in CDS immediately after smoking cannabis with a gradual return to expected CDS levels afterwards. Cohen's d will be used as an estimate for the effect size for measuring the difference in changes in CDS from baseline (pre-cannabis) to the time point with the assumed largest differences between the two groups. Under these assumptions, power for finding a significant difference in changes in CDS between the 13.4% THC group and the placebo was estimated using 1000 simulations, which showed 80% power to detect Cohen's $d=0.33$ or larger with significance level $\alpha=0.05$. The estimates suggest adequate power to detect likely changes associated with cannabis smoking. For example, in our previous study participants smoked cigarettes with 4% THC, and 2 to 3 hours post-smoking evidenced effect sizes between 0.36 and 0.47 when comparing changes in SDLP between placebo and active THC.

HUMAN SUBJECTS

Total number of participants to be enrolled: We will recruit 240 potential participants to eventually enroll 180 participants who meet inclusion/exclusion criteria and provide complete data. Eligible participants will return for one experimental visit to receive one of the three types of cannabis to be evaluated.

Age: participants must be aged 21-55 (upper limit is to minimize potential confounding by medical conditions associated with aging). Because of the problems inherent in the use of cannabis in children and adolescents, we will not enroll individuals below the age of 21.

Gender: Both males and females will be recruited.

Ethnic background: Given the diverse ethnic background of San Diego (see Figure 2 below), we should be able to recruit subjects from multiple ethnic backgrounds.

Figure 2

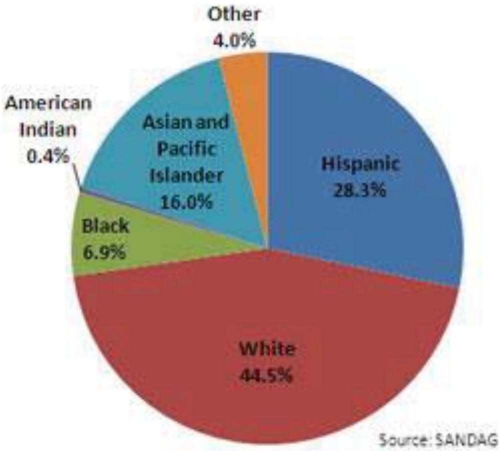

Inclusion criteria

- Age greater than 21
- Must be a licensed driver and driven a minimum of 1,000 miles in the past year
- Must be a regular cannabis smoker (>/=4 times in the past month)
- Willing to not disclose details of the simulator and iPad based assessments

Exclusion criteria

- History of traumatic brain injury.
- At the discretion of the examining physician, individuals with significant cardiovascular, hepatic or renal disease, uncontrolled hypertension, and chronic pulmonary disease (e.g., asthma, COPD) will be excluded.
- Unwillingness to abstain from cannabis for:
    - 2 days prior to screening visit (so driving simulation will not be impaired)
    - 2 days prior to experimental visit (2-3 half-lives of THC)
- Positive pregnancy test
- A positive result on toxicity screening for cocaine, amphetamines, opiates, and phencyclidine (PCP) will exclude individuals from participation. However, a positive result for a prescribed or recommended medication (cannabis) will not be exclusionary.
- Substance Abuse History: Individuals with current substance use disorders[57] as assessed using the Drug Abuse Screening Test (DAST) and Alcohol Use Disorders Identification Test (AUDIT).
- Schizophrenia, Bipolar Disorder with a history of mania, other psychotic disorder, current suicidal ideation or past history of suicide attempt.
- Suicidality. The Beck Depression Inventory-II (BDI-II) will be administered during the in-person screening evaluation. Participants will be excluded from the study if their BDI-II score is greater than or equal to 17 or if suicidal ideas are endorsed on the BDI-II assessments. Community referrals will be made when appropriate.
- Must be willing to be transported by cab or have a friend/family member drive them home after experimental session
- Inability to complete study procedures (i.e. poor veins, unwillingness to be transported home by taxi or friend)

Recruitment and Procedures Preparatory to Research

Subjects will be recruited from the community. CMCR outreach personnel will maintain a presence at many community events, and give presentations at support and other services groups.

Methods to identify and recruit research study participants: include an education campaign utilizing newspaper advertisements; a system to promote referrals from health care providers, case managers, and service agency staff; and direct contact with potential participants via outreach conducted in community settings, clinics, and hospital venues. Educational materials are distributed both through traditional (e.g., newsletters, newspapers, community based organizations, doctor's offices and medical clinics) and "non-traditional" (e.g., Craig's list, bookstores, pharmacies, nutritionists, massage therapists, and social organizations) venues. Referral networks are built and maintained though community meetings, events, and activities. Outreach is conducted via informational tables at health fairs, community events and community venues including physician offices and medical clinics.

Recruitment will also occur through the CMCR where participants are given the option on their consents to be contacted for future studies. We will only contact those individuals who have consented to be contacted for future studies on their CMCR-affiliated consent document – or who have signed a screening consent for a CMCR-affiliated IRB approved project.

We will add an online survey using the Platinum Edition of Survey 2 Monkey to screen potential subjects. The Platinum Edition of Survey Monkey is HIPAA compliant – please see https://www.surveymonkey.com/pricing/details/.

Pre-Screen Phone Call

Volunteers will be screened via telephone interview and, as appropriate, via face-to-face assessment. Telephone screening (respondents blind to selection criteria) will assure volunteers meet general age and medical criteria.

Procedures Preparatory To Research During the start-up phase we will establish key infrastructure components, as well as develop the assessment tools needed to initiate the clinical research. These include:

a) IND 131268 for the use of cannabis in this study was approved by the Food and Drug Administration (FDA). The National Institute of Drug Abuse (NIDA) provided a Letter of Authorization for the FDA to view their drug supply program Master Drug File and a letter acknowledging that they would supply the cannabis for this protocol. Approval from the DEA is pending UCSD IRB approval. This is now in progress. The Regulatory Panel of California has approved the protocol.
b) Training of staff.
c) Development/refinement of Δ9-THC assays. To ensure that analytical measurements of Δ9-THC and metabolites are accurate, precise, and reproducible using isotope dilution ultra-performance liquid chromatography (UPLC) and tandem mass spectrometry (MS/MS), we will conduct initial studies targeting (−)-trans-Δ9-tetrahydrocannabinol (THC), 11-hydroxy-Δ9-tetrahydrocannabinol (11-OH-THC), 11-nor-9-carboxy-delta-9-tetrahydrocannabinol (THC-COOH) and cannabidiol (CBD) in whole blood. The target limit of quantification (LOQ) will be 0.5 ng/mL of each of the components in whole blood.
d) Driving simulation development. This includes purchasing and setting up simulator hardware, as well as modifying simulations to best assess the types of driving-related difficulties likely to occur when under the influence of THC.
e) Development of a suite of tablet-based tests for a potential performance-based field sobriety test. Based upon the extant literature regarding the cognitive effects of Δ9-THC and impact on driving, we will develop a suite of approximately 5 tests as potential performance-based measures of impaired functioning for use in the field. Data generated by the clinical research will inform future decisions regarding which tests are the most sensitive to such impairments.

Informed Consent

Informed consent will be obtained from all individuals participating in this study. All recruiters at the CMCR have tremendous experience with the informed consent process and sensitivity to the impairments that may be associated with substance use and psychiatric disorders. Recruiters who have undergone CITI and HIPAA training will explain the study to potential participants. They will have sufficient knowledge of the study to answer any questions regarding the study. They will explain the research activity, how it is experimental (e.g.,

a new drug, extra tests, separate research records, or nonstandard means of management, such as flipping a coin for random assignment or other design issues). They will inform the human subjects of the reasonably foreseeable harms, discomforts, inconvenience and risks that are associated with the research activity. During the informed consent procedure, patients will be informed that all data obtained in the interviews is strictly confidential, and that no information will be shared with others without the participant's express written approval. To enhance comprehension, the informed consent documents are written at the 8th grade level of language. Written informed consent will be obtained from each participant prior to enrollment in the study. No individuals from vulnerable populations will be recruited. Participants will be given a copy of the consent document, as well as the "Experimental Subject's Bill of Rights" to keep.

The informed consent document will contain a section informing the subjects that by signing the consent, they are agreeing that data collected (e.g., cognitive tests, interviews, questionnaires, plasma, saliva) in other IRB-approved CMCR studies that they may be enrolled in may be used in this study.

Similarly, the informed consent document will include a statement informing subjects that data and samples gathered in this study may be shared with other CMCR investigators conducting IRB-approved research. Since future research using banked samples from this study could include genetic analysis the appropriate language from the DNA and Informed Consent Fact Sheet has been included in the consent.

Additionally, Protected Health Information (PHI) will not be obtained without a separate "Authorization to Obtain Medical Records" consent.

Potentials volunteers will be pre-screened via telephone interview by members of the PAR and, as appropriate, via face-to-face assessment by a clinician at the Screening Interview. Telephone screening (respondents blind to selection criteria) will assure volunteers meet general age and medical criteria.

For telephone pre-screening, we are applying for Waiver of Documented Consent. We will obtain oral consent. The pre-screening interview presents no more than minimal risk of harm to subjects. Data collected during the pre-screen will be used for the purposes of determining eligibility and for comparing the characteristics of participants who were enrolled in the study to those who were deemed ineligible. Participant identifiers collected during pre-screen will be retained so that any recruiter who speaks with the individual will have access to the information. Identifying information is stored encrypted on a physically segregated internal network with absolutely no links to the de-identified scientific data. Only select staff members have access to this database.

A signed main consent form and HIPAA form will be obtained at the Screening Interview.

Potential Risks

Likely

- lethargy
- difficulties with balance
- eye irritation
- throat irritation
- increased heart rate
- possible low blood pressure
- reversible problems with your appetite
- slight nausea or queasiness from the driving simulation

Less Likely

- dizziness
- some change in your mood (good or bad)
- loss of memory

- decreased ability to concentrate or think properly
- nausea to the point of vomiting, from the driving simulation

Rare But Serious
- head and chest pressure
- disorientation
- agitation
- combativeness
- incoherence
- visual hallucinations
- panic attacks
- fainting

All of the above are potentially less likely when randomized to placebo.

Physical harm: Risks of inhaled cannabis products may include psychomotor coordination difficulties, eye irritation, throat irritation, increased heart rate, possible hypotension, and reversible appetite/mood/memory/cognition effects.

There may be some discomfort when blood samples are taken, and there is a small risk of bruising, infection, or inflammation at the site at which the needle is inserted. We will be taking up to approximately 21 teaspoons of blood for the purposes of this study.

- **Psychological harm:** anxiety and/or emotional distress may result from questions asked during assessment or as a result of the time taken in the assessment process. Additionally, some iPad tests may require concentrated effort and may be frustrating for the subject to complete.
- **Legal harm:** We will be asking sensitive questions about use of cannabis. Access to such material for legitimate research purposes is generally acceptable, as long as the researcher protects the confidentiality of that information. We will use all available methods to ensure confidentiality, including a Certificate of Confidentiality from the National Institute of Drug Abuse.
- **Social harm:** Invasions of privacy and breaches of confidentiality may result in embarrassment within one's business or social group. Every effort will be made to maintain confidentiality of the subject's participation to lessen this type of risk.
- **Economic harm:** Eligibility for insurance, political campaigns, and standing in the community are problems may result from loss of confidentiality. Smoking marijuana may hinder application for future employment, if drug screening is a condition of employment. It is likely that detectable traces of marijuana will remain in the subject's hair or blood for a minimum of six weeks after smoking marijuana. If applicable, a letter will be written to the subject's employer explaining their participation in this research study and the dates of participation.
- **Reproductive risks:** The procedures in this research are known to hurt a pregnancy or fetus in the following ways: poor educational attainment. A participant should not become pregnant or father a baby while on this study because the drugs in this study can affect an unborn baby. Women should not breastfeed a baby while on this study. Subjects will be advised they should use birth control while on this study if they engage in opposite sex relations and have not undergone sterilization procedures (vasectomy, tubal ligation). Acceptable methods of birth control are: oral contraceptive pills, diaphragm and condom with spermicide, progestin implant or injection, intrauterine contraceptive device and abstinence.
- **Unknown Risks:** The experimental treatments may have side effects that no one knows about yet. The researchers will let subjects know if they learn anything that might make you change your mind about participating in the study.
- **Breach of Confidentiality**: One potential risk is that of breach of confidentiality wherein a person's DNA information (genetic risk for certain diseases), drug use history, or other sensitive information might be disclosed, resulting in embarrassment or even prejudicial treatment by others.

Risk Management Procedures and Adequacy of Resources

Risk Management Procedures

In order to minimize the risk of simulator sickness, participants will be slowly trained to adapt to the driving simulator. The room will also be kept at a cool temperature, and participants will have a small fan blowing on them. Participants will be evaluated with a Simulator Sickness Questionnaire upon completion of the driving simulations (assesses symptoms of headache, dizziness, nausea, etc.). Any concerning symptoms will be addressed by the examiner, and if necessary, a clinician. In the event that any participant does experience simulator sickness during the course of the study, they will be offered the opportunity to take a break, lie down on an examining table with the room lights dimmed. If the sickness continues, they will be offered the opportunity to discontinue the study.

Vital signs will be monitored throughout the experiment to monitor the subject's health status as well as to quantify marijuana's general effects. At any sign of an adverse reaction (e.g. a change in blood pressure or pulse rate or development of psychological distress), an investigator will be called. Subjects can be transported to the emergency room. Subjects will remain in the laboratory under direct observation for 6 hours after the marijuana smoking inhalations are completed. At that time, a final vital sign and self-report status check will be made and upon satisfactory readings, the subject will be released and driven back to his/her domicile by taxicab or prearranged transportation. The return transport procedure also will be observed directly by staff to ensure compliance.

To insure safety after the inhalation of the study drug, participants will not be allowed to drive themselves home. They must appoint a designated driver or if they cannot, we will arrange for taxi service. Participants will be counseled that they are not to drive or operate heavy machinery the day of the study, should they leave the study visit early.

To reduce fatigue, the time needed to complete the cognitive and behavioral interviews will be minimized by familiarizing the interviewers with the contents of the questionnaires. Our interviewers are trained not to press participants to answer questions that seem to be excessively distressing to them, and interviews will be terminated if the participant is too distressed, too fatigued, or excessively frustrated by the effort.

To minimize the risk of hunger and/or dehydration, snacks and juices will be provided throughout the day.

A HNRP/CMCR clinician (psychologists, psychiatrists or Masters-level clinician) will be consulted and will make an assessment in the event that an individual becomes distressed during the course of the interviews, the Beck Depression Inventory-II score is greater than or equal to 17, or suicidal ideas are endorsed on the Beck assessments. This assessment involves a semi-structured interview to determine whether the participant is an immediate danger (i.e. suicidal ideation with intent to harm). If the participant is not in immediate danger, they will be provided with a list of mental health resources. If the participant does appear to be in immediate danger, the psychologist will determine the participant's willingness to be assessed in the Emergency Department. If the participant is willing, the psychologist will escort the participant to the Hillcrest UCSD Medical Center Emergency Department. If not willing, the psychologist will call Campus emergency, or 911 if the participant leaves the premises. Community referrals will be made when appropriate.

Data and Safety Monitoring Board (DSMB)

A DSMB will be selected utilizing a group of experts that will advise the study investigators, with the primary responsibility to monitor human subject safety. The members will be comprised of at least 3 independent clinicians familiar with the conduct of clinical trials. The DSMB will track treatment, laboratory results, clinical assessments and any adverse events.

DSMB meetings may take place via online meeting if not every member is available to meet in person at each timepoint. The study's statistician will prepare an open report available to all essential members of the study and the DSMB members and a closed report reviewed only by the DSMB members. The open report will

present data in aggregated form (not separated by arm). The closed report will present data by treatment arm, but in blinded fashion, i.e. the arms will be randomly labeled as arm A and arm B. Treatment labels can be revealed to the DSMB members upon their request if major safety concerns arise.

The reports will include the following data:

(1) Enrollment and study status: the number of 1) screened subjects; 2) exclusions and reasons; 3) enrolled subjects and projected enrollment; 4) subjects by visit; 5) subjects completing the entire study; 6) subjects who withdrew (drop-outs) from the study and reasons; and 7) missing visits and reasons.
(2) Demographic and relevant clinical characteristics of the cohort
(3) Lab values by visit
(4) Vital signs and outcomes of medical exam
(5) Safety data and study related adverse events: 1) the number of adverse events; 2) type and severity (grade) of the adverse events (mild, moderate, severe), as well as duration and the outcome of adverse events; 3) number of subjects with adverse events; 4) number of deaths related to adverse events; 5) unanticipated problems.
(6) Protocol deviations

The members of the DSMB will be asked to maintain confidentiality related to the interim data presented in the closed report until the end of the trial.

Privacy and Confidentiality Considerations Including Data Access and Management
The CMCR has stringent protocols in place to protect the privacy of participants and the confidentially of data. Specifically, participant-derived data and samples are de-identified, assigned a coded ID, and are maintained according to a standardized, confidential, and secure manner. Per CMCR standard policy, strict confidentiality will be maintained. All members of the investigative team are trained regarding the protection of participants' rights to confidentiality. The investigative teams is required to successfully complete training according to standards of the Health Insurance Portability and Accountability Act, and to complete the UCSD certification requirements. The systems within the research center comply with HIPAA regulations for protection of person identifiable health data. To ensure confidentiality, only the participant's code number appears on all of the data and forms. Any identifiable information (PII) within the research center is stored encrypted on a physically segregated internal network with absolutely no links to the de-identified scientific data. The data is stored on a server located within a keycard restricted server room with extremely limited physical access. In addition to the physical restrictions, these clients are authenticated against MAC address and a username/password challenge. The two data systems (identifiable network, de-identified research information system) utilize separate Access Control Lists (ACL).

Each sample is labeled with a unique sample specific ID. The data linking these sample IDs to their corresponding non-identifiable study ID is stored in a segregated secure database. All stored samples are accessible only to the CMCR laboratory personnel and the appropriate study members. Samples are stored under the coded identifiers in freezers equipped with locks. In addition, these freezers are located behind locked doors that require ID scan entry.

To help protect the privacy of subjects, the investigators have obtained a Confidentiality Certificate from the National Institute on Mental Health (NIMH). With this Certificate, the investigators cannot be forced by court subpoena to disclose information that may identify a subject in any federal, state, or local civil, criminal, administrative, legislative, or other proceedings. Disclosure will be necessary, however, upon request of DHHS or the UCSD Human Research Protections Program for the purpose of audit or evaluation.

Potential Benefits
There is no direct benefit to subjects.

Risk/Benefit Ratio

There was a recent safety study of a “standardized herbal cannabis product with 12.5% Δ9-THC” conducted at seven pain clinics over a one year period[58]. This was a similar concentration to the highest concentration that we will be using during our 8-hour human laboratory experiment. Controls in the safety study were individuals with chronic pain from the same clinics who were not cannabis users. The primary outcome consisted of serious adverse events and non-serious adverse events. Secondary safety outcomes included pulmonary and neurocognitive function and standard hematology, biochemistry, renal, liver, and endocrine function. Secondary efficacy parameters included pain and other symptoms, mood, and quality of life. There was no difference in risk of serious adverse events (adjusted incidence rate ratio = 1.08, 95% confidence interval = .57–2.04) between groups. Medical cannabis users were at increased risk of non-serious adverse events (adjusted incidence rate ratio = 1.73, 95% confidence interval = 1.41–2.13); most were mild to moderate. There were no differences in secondary safety assessments. The authors reasoned that herbal cannabis, when used by patients with experience of cannabis use as part of a monitored treatment program over 1 year, appears to have a reasonable safety profile.

The present study differs in that participants will be given cannabis acutely. Acute effects may include anxiety and panic, impaired attention, and memory (while intoxicated), and an increased risk of psychotic symptoms. Short term cannabis intoxication can hinder the mental processes of organizing and collecting thoughts[59]. Psychotic episodes are well-documented and typically resolve within minutes or hours although there have been few reports of symptoms lasting longer[60]. Cannabis has not been reported to cause fatal overdose[61]. The other major difference is that there are no benefits (e.g., pain relief) to be provided the volunteers in the present study.

The investigators in the present study have performed previous clinical trials involving acute cannabis exposure[62,63]. Cannabis was well tolerated other than there being psychoactive effects and some memory impairment acutely. We believe the risk/benefit ratio of the present study to be favorable in the context of the knowledge to be gained and the public health peril of driving under the influence of cannabis.

Expense to Participant

There will no expense for participants.

Compensation for Participation

Subject payments are requested in order to compensate subjects for their participation. Participants will be asked to arrange for transportation to and from the research site. If this is not feasible, a taxi ride will be arranged for them. We will pay subjects for the driving simulation performed during the screening visit a payment of $50. We will compensate subjects for time and trouble during the experimental visit at $22.50 per hour times 8 hours equals $180 per subject (potential total of $230). Full compensation will be given once we have confirmation that a participant has a reliable form of transportation home (i.e. taxi service or friend). Compensation will be pro-rated if the subject does not complete the visit at $22.50 per hour. Subjects who begin but do not complete the screening visit (e.g. due to ineligibility) will be provided $10.

FUNDING SUPPORT

This study was authorized and will be funded by the California Legislature pursuant to Assembly Bill 266 (Bonta/Cooley/Jones-Sawyer/Lackey), the Medical Marijuana Regulation and Safety Act[1].

DRUG PROCESSING

Given that we have received IND approval from the FDA, investigational drugs will be prepared for this study by the UCSD Investigational Pharmacy. Accountability records will been maintained according to policies and procedures.

Sign out logs will be kept as dictated by DEA officials. At the end of each experimental session and/or study visit, all unused materials will be collected and stored in a sealed container that will be returned to the UCSD Investigational Pharmacy, with the exact amount noted and dated in the log (e.g., “bulk cannabis weighing x mg”). All records will be made available to the DEA and the Research Advisory Panel of California, which supervises all controlled substance research in California. At the end of the study, all unused plant material

(i.e., cannabis material not smoked or the incinerated product from cigarette combustion) from each subject's driving simulation session will be collected and placed in a container, which will disposed at the facility used to incinerate unwanted medical materials.

BIBLIOGRAPHY

1. AB-266 Medical marijuana.(2015-2016) https://leginfo.legislature.ca.gov/faces/billNavClient.xhtml?bill_id=201520160AB266. Accessed.
2. Grotenhermen F, Leson G, Berghaus G, et al. Developing limits for driving under cannabis. *Addiction (Abingdon, England).* 2007;102(12):1910-1917.
3. Ramaekers JG, Berghaus G, van Laar M, Drummer OH. Dose related risk of motor vehicle crashes after cannabis use. *Drug Alcohol Depend.* 2004;73(2):109-119.
4. Drummer OH, Gerostamoulos J, Batziris H, et al. The involvement of drugs in drivers of motor vehicles killed in Australian road traffic crashes. *Accid Anal Prev.* 2004;36(2):239-248.
5. Hartman RL, Huestis MA. Cannabis Effects on Driving Skills. *Clin Chem.* 2013;59(3):478-492.
6. Skopp G, Potsch L. Cannabinoid concentrations in spot serum samples 24-48 hours after discontinuation of cannabis smoking. *J Anal Toxicol.* 2008;32(2):160-164.
7. Sewell RA, Poling J, Sofuoglu M. The effect of cannabis compared with alcohol on driving. *Am J Addict.* 2009;18(3):185-193.
8. Kelly E, Darke S, Ross J. A review of drug use and driving: epidemiology, impairment, risk factors and risk perceptions. *Drug Alcohol Rev.* 2004;23(3):319-344.
9. Bates MN, Blakely TA. Role of cannabis in motor vehicle crashes. *Epidemiol Rev.* 1999;21(2):222-232.
10. Walsh JM, de Gier JJ, Christopherson AS, Verstraete AG. Drugs and driving. *Traffic Inj Prev.* 2004;5(3):241-253.
11. Papafotiou K, Carter JD, Stough C. An evaluation of the sensitivity of the Standardised Field Sobriety Tests (SFSTs) to detect impairment due to marijuana intoxication. *Psychopharmacology (Berl).* 2005;180(1):107-114.
12. Bosker WM, Theunissen EL, Conen S, et al. A placebo-controlled study to assess Standardized Field Sobriety Tests performance during alcohol and cannabis intoxication in heavy cannabis users and accuracy of point of collection testing devices for detecting THC in oral fluid. *Psychopharmacology (Berl).* 2012;223(4):439-446.
13. Hart CL, van Gorp W, Haney M, Foltin RW, Fischman MW. Effects of acute smoked marijuana on complex cognitive performance. *Neuropsychopharmacology.* 2001;25(5):757-765.
14. Ramaekers JG, Theunissen EL, de Brouwer M, Toennes SW, Moeller MR, Kauert G. Tolerance and cross-tolerance to neurocognitive effects of THC and alcohol in heavy cannabis users. *Psychopharmacology (Berl).* 2011;214(2):391-401.
15. Huestis MA, Verstraete A, Kwong TC, Morland J, Vincent MJ, de la Torre R. Oral fluid testing: promises and pitfalls. *Clin Chem.* 2011;57(6):805-810.
16. Huestis MA, Henningfield JE, Cone EJ. Blood cannabinoids. II. Models for the prediction of time of marijuana exposure from plasma concentrations of delta 9-tetrahydrocannabinol (THC) and 11-nor-9-carboxy-delta 9-tetrahydrocannabinol (THCCOOH). *Journal of Analytical Toxicology.* 1992;16(5):283-290.
17. Fabritius M, Chtioui H, Battistella G, et al. Comparison of cannabinoid concentrations in oral fluid and whole blood between occasional and regular cannabis smokers prior to and after smoking a cannabis joint. *Analytical and bioanalytical chemistry.* 2013;405(30):9791-9803.
18. Perez-Reyes M. Marijuana smoking: factors that influence the bioavailability of tetrahydrocannabinol. *NIDA Res Monogr.* 1990;99:42-62.
19. Himes SK, Scheidweiler KB, Beck O, Gorelick DA, Desrosiers NA, Huestis MA. Cannabinoids in exhaled breath following controlled administration of smoked cannabis. *Clin Chem.* 2013;59(12):1780-1789.
20. Carter GT, Weydt P, Kyashna-Tocha M, Abrams DI. Medicinal cannabis: rational guidelines for dosing. *IDrugs.* 2004;7(5):464-470.

21. Brookhuis KA, Volkerts ER, O'Hanlon JF. Repeated dose effects of lormetazepam and flurazepam upon driving performance. *Eur J Clin Pharmacol.* 1990;39(1):83-87.
22. Ramaekers JG, O'Hanlon JF. Acrivastine, terfenadine and diphenhydramine effects on driving performance as a function of dose and time after dosing. *Eur J Clin Pharmacol.* 1994;47(3):261-266.
23. Theunissen EL, Vermeeren A, van Oers AC, van Maris I, Ramaekers JG. A dose-ranging study of the effects of mequitazine on actual driving, memory and psychomotor performance as compared to dexchlorpheniramine, cetirizine and placebo. *Clin Exp Allergy.* 2004;34(2):250-258.
24. Weiler JM, Bloomfield JR, Woodworth GG, et al. Effects of fexofenadine, diphenhydramine, and alcohol on driving performance: A randomized placebo-controlled trial in the Iowa Driving Simulator. *Annals of Internal Medicine.* 2000;132:354-363.
25. Lenne MG, Dietze P, Rumbold GR, Redman JR, Triggs TJ. The effects of the opioid pharmacotherapies methadone, LAAM and buprenorphine, alone and in combination with alcohol, on simulated driving. *Drug Alcohol Depend.* 2003;72(3):271-278.
26. Arnedt JT, Wilde GJ, Munt PW, MacLean AW. How do prolonged wakefulness and alcohol compare in the decrements they produce on a simulated driving task? *Accid Anal Prev.* 2001;33(3):337-344.
27. Ramaekers JG, Robbe HW, O'Hanlon JF. Marijuana, alcohol and actual driving performance. *Hum Psychopharmacol.* 2000;15(7):551-558.
28. Brookhuis KA, de Waard D, Samyn N. Effects of MDMA (ecstasy), and multiple drugs use on (simulated) driving performance and traffic safety. *Psychopharmacology.* 2004;173(3-4):440-445.
29. Marcotte TD, Heaton RK, Wolfson T, et al. The impact of HIV-related neuropsychological dysfunction on driving behavior. *Journal of the International Neuropsychological Society.* 1999;5(7):579-592.
30. Marcotte TD, Rosenthal TJ, Roberts E, et al. The contribution of cognition and spasticity to driving performance in multiple sclerosis. *Archives of physical medicine and rehabilitation.* 2008;89(9):1753-1758.
31. O'Hanlon JF. Driving performance under the influence of drugs: rationale for, and application of, a new test. *British journal of clinical pharmacology.* 1984;18 Suppl 1:121S-129S.
32. O'Hanlon JF, Volkerts ER. Hypnotics and actual driving performance. *Acta Psychiatr Scand Suppl.* 1986;332:95-104.
33. Vermeeren A, O'Hanlon JF. Fexofenadine's effects, alone and with alcohol, on actual driving and psychomotor performance. *The Journal of allergy and clinical immunology.* 1998;101(3):306-311.
34. Marcotte TD, Scott JC. The assessment of driving abilities. *Advances in Transportation Studies: An International Journal.* 2004:79-90.
35. Ostlund J, Nilsson L, Tomros J, Forsman A. *Calibration tasks for methods which assess demand due to the use of invehicle systems.* Linkoping, Sweden: VTI;2006.
36. Engelmann C. California Manual on Uniform Traffic Control Devices (MUTCD). Sacramento, CA: State of California. http://www.dot.ca.gov/hq/traffops/engineering/mutcd/. In: (Caltrans) CDoT, ed2014.
37. Simons-Morton BG, Bingham CR, Falk EB, et al. Experimental effects of injunctive norms on simulated risky driving among teenage males. *Health Psychol.* 2014;33(7):616-627.
38. Caird J, Chisholm S, Edwards C, Creaser J. The effect of yellow light onset time on older and younger drivers' perception response time (PRT) and intersection behavior. *Transportation Research.* 2007;10(Part F):383-396.
39. Ramaekers JG, Kauert G, Theunissen EL, Toennes SW, Moeller MR. Neurocognitive performance during acute THC intoxication in heavy and occasional cannabis users. *J Psychopharmacol.* 2009;23(3):266-277.
40. Bosker WM, Karschner EL, Lee D, et al. Psychomotor function in chronic daily Cannabis smokers during sustained abstinence. *PLoS One.* 2013;8(1):e53127.
41. Ramaekers JG, Moeller MR, van Ruitenbeek P, Theunissen EL, Schneider E, Kauert G. Cognition and motor control as a function of Delta9-THC concentration in serum and oral fluid: limits of impairment. *Drug Alcohol Depend.* 2006;85(2):114-122.
42. Sewell RA, Schnakenberg A, Elander J, et al. Acute effects of THC on time perception in frequent and infrequent cannabis users. *Psychopharmacology (Berl).* 2013;226(2):401-413.
43. Miller LL, McFarland D, Cornett TL, Brightwell D. Marijuana and memory impairment: effect on free recall and recognition memory. *Pharmacol Biochem Behav.* 1977;7(2):99-103.

44. Ranganathan M, D'Souza DC. The acute effects of cannabinoids on memory in humans: a review. *Psychopharmacology (Berl).* 2006;188(4):425-444.
45. Lane SD, Cherek DR, Lieving LM, Tcheremissine OV. Marijuana effects on human forgetting functions. *J Exp Anal Behav.* 2005;83(1):67-83.
46. Wolff K, Johnston A. Cannabis use: a perspective in relation to the proposed UK drug-driving legislation. *Drug Test Anal.* 2014;6(1-2):143-154.
47. Chindarkar NS, Park HD, Stone JA, Fitzgerald RL. Comparison of Different Time of Flight-Mass Spectrometry Modes for Small Molecule Quantitative Analysis. *J Anal Toxicol.* 2015;39(9):675-685.
48. Schwope DM, Karschner EL, Gorelick DA, Huestis MA. Identification of recent cannabis use: whole-blood and plasma free and glucuronidated cannabinoid pharmacokinetics following controlled smoked cannabis administration. *Clin Chem.* 2011;57(10):1406-1414.
49. JK H, JC T, PG M. Cannabinoid content of individual plant organs from different geographical strains of Cannabis sativa L. *Journal of natural products.* 1980;43:112-122.
50. ElSohly M. Chem constituents of cannabis. In: Grotenhermen F, Russo E, eds. *Cannabis and cannabinoids: pharmacology, toxicology, and therapeutic potential.* New York: Haworth Integrative Healing Press; 2002:27-36.
51. Pomahacova B, Van der Kooy F, Verpoorte R. Cannabis smoke condensate III: the cannabinoid content of vaporised Cannabis sativa. *Inhal Toxicol.* 2009;21(13):1108-1112.
52. Elsohly HN, Elsohly MA. Marijuana smoke condensate: chemistry and pharmacology. In: ElSohly M, ed. *Marijuana and the cannabinoids.* Totowa (NJ): Humana Press; 2007:67-96.
53. Moore C, Vincent M, Rana S, Coulter C, Agrawal A, Soares J. Stability of Delta(9)-tetrahydrocannabinol (THC) in oral fluid using the Quantisal collection device. *Forensic Sci Int.* 2006;164(2-3):126-130.
54. Toennes SW, Kauert GF, Steinmeyer S, Moeller MR. Driving under the influence of drugs -- evaluation of analytical data of drugs in oral fluid, serum and urine, and correlation with impairment symptoms. *Forensic Sci Int.* 2005;152(2-3):149-155.
55. Samyn N, De Boeck G, Wood M, et al. Plasma, oral fluid and sweat wipe ecstasy concentrations in controlled and real life conditions. *Forensic Sci Int.* 2002;128(1-2):90-97.
56. Beck O, Stephanson N, Sandqvist S, Franck J. Detection of drugs of abuse in exhaled breath using a device for rapid collection: comparison with plasma, urine and self-reporting in 47 drug users. *Journal of breath research.* 2013;7(2):026006.
57. Robins LN, Helzer JE, Croughan J, Ratcliff KS. National Institute of Mental Health Diagnostic Interview Schedule. Its history, characteristics, and validity. *Arch Gen Psychiatry.* 1981;38(4):381-389.
58. Ware MA, Wang T, Shapiro S, Collet JP. Cannabis for the Management of Pain: Assessment of Safety Study (COMPASS). *J Pain.* 2015;16(12):1233-1242.
59. Hall W, Solowij N. Adverse effects of cannabis. *Lancet.* 1998;352(9140):1611-1616.
60. D'Souza DC, Sewell RA, Ranganathan M. Cannabis and psychosis/schizophrenia: human studies. *Eur Arch Psychiatry Clin Neurosci.* 2009;259(7):413-431.
61. Calabria B, Degenhardt L, Hall W, Lynskey M. Does cannabis use increase the risk of death? Systematic review of epidemiological evidence on adverse effects of cannabis use. *Drug Alcohol Rev.* 2010;29(3):318-330.
62. Wilsey B, Marcotte T, Tsodikov A, et al. A randomized, placebo-controlled, crossover trial of cannabis cigarettes in neuropathic pain. *J Pain.* 2008;9(6):506-521.
63. Wilsey B, Marcotte T, Deutsch R, Gouaux B, Sakai S, Donaghe H. Low-dose vaporized cannabis significantly improves neuropathic pain. *J Pain.* 2013;14(2):136-148.
