# Supplementary material for: Evaluation of Field Sobriety Tests for Identifying Drivers Under the Influence of Cannabis: A Randomized Clinical Trial
Source: JAMA Psychiatry. 2023 Aug 2;80(9):914–23. doi: 10.1001/jamapsychiatry.2023.2345 (PMC10398547; doi:10.1001/jamapsychiatry.2023.2345)
Supplement: Supplement 2. — eTable 1. Field Sobriety Tests Used in the Current Study eTable 2. Percentage of Participants Classified as FST-Impaired, by Officer Estimation Regarding Treatment Assignment eTable 3. Percentage of Participants Classified as FST-Impaired, by What the Officer Believed Was the Person’s Treatment Assignment eTable 4. Percentage of Participants Showing an FST Clue at Each Time Point by Treatment Arm Among Those Classified as FST-Impaired at the First FST Evaluation eTable 5. Association Between Demographic Characteristics and Officer-Determined FST Impairment at 71 Minutes After Smoking in the Placebo Group eTable 6. Univariable Associations Between FST Items and Being Classified as FST-Impaired by the Officer in the Placebo Group eAppendix. Driving Simulations, Randomization and Blinding, Power and Sample Size, and Adverse Events eReferences [file jamapsychiatry-e232345-s002.pdf]

Supplementary Online Content

Marcotte TD, Umlauf A, Grelotti DJ, et al. Evaluation of field sobriety tests for identifying drivers under the influence of cannabis: a randomized clinical trial. *JAMA Psychiatry*. Published online August 2, 2023. doi:10.1001/jamapsychiatry.2023.2345

eTable 1. Field Sobriety Tests Used in the Current Study
eTable 2. Percentage of Participants Classified as FST-Impaired, by Officer Estimation Regarding Treatment Assignment
eTable 3. Percentage of Participants Classified as FST-Impaired, by What the Officer Believed Was the Person's Treatment Assignment
eTable 4. Percentage of Participants Showing an FST Clue at Each Time Point by Treatment Arm Among Those Classified as FST-Impaired at the First FST Evaluation
eTable 5. Association Between Demographic Characteristics and Officer-Determined FST Impairment at 71 Minutes After Smoking in the Placebo Group
eTable 6. Univariable Associations Between FST Items and Being Classified as FST-Impaired by the Officer in the Placebo Group
eAppendix. Driving Simulations, Randomization and Blinding, Power and Sample Size, and Adverse Events
eReferences

This supplementary material has been provided by the authors to give readers additional information about their work.

© 2023 Marcotte TD et al. *JAMA Psychiatry.*

eTable 1. Field sobriety tests used in the current study

Test	Brief Summary
Walk-and-Turn (WAT)	Participants are to take nine steps, touching heel-to-toe, along a straight line. After taking the steps, participants must turn on one foot and return in the same manner in the opposite direction.
One Leg Stand (OLS)	Participants are to stand with one foot approximately six inches off the ground and count aloud by ones beginning with one thousand (one thousand-one, one thousand-two, etc.) until told to put the foot down. The officer times the participant for 30 seconds.
Finger-to-Nose (FTN)	Participants are to close their eyes, tilt their head back slightly, and touch their nose with their index finger. The commands for the participant's index fingers are given in this sequence: Left, Right, Left, Right, Right, Left. After the sixth attempt, participants are instructed to open their eyes.
Lack of Convergence (LOC)	Participants are to watch an object (e.g., an eraser on a pencil) that is moved towards the bridge of their nose until it is approximately 1 inch from the bridge. During this movement, the officer watches the eyes, which should come together and then converge/cross. Officers also determined pupillary diameter.
Modified Romberg Test (MROM)	Participants are to stand with their feet together, head tilted backward with eyes closed, and notify the examiner when the participant believes that 30 seconds have passed.

© 2023 Marcotte TD et al. *JAMA Psychiatry.*

eTable 2. Percentage of participants classified as FST-impaired, by officer estimation regarding treatment assignment.

Officer belief of which Tx participant received:	Officer determination of FST-impairment		Number of participants
	Not impaired	FST-Impaired	
THC (Strongly)	1 (1.0%)	95 (99.0%)	**96**
THC (Somewhat)	9 (22.0%)	32 (78.0%)	**41**
Do not know	5 (100.0%)	0 (0.0%)	**5**
Placebo (Somewhat)	16 (94.1%)	1 (5.9%)	**17**
Placebo (Strongly)	23 (100.0%)	0 (0.0%)	**23**

© 2023 Marcotte TD et al. *JAMA Psychiatry.*

eTable 3. Percentage of participants classified as FST-impaired by the officer, by what the officer believed (strongly believed, somewhat believed, don't know) was the person's treatment assignment.

Officer belief regarding which Tx participant received:	Officer determination of FST-impairment	
	Not impaired	FST-Impaired
THC (Strongly)	1 (1.9%)	95 (74.2%)
THC (Somewhat)	9 (16.7%)	32 (25.0%)
Do not know	5 (9.3%)	0 (0.0%)
Placebo (Somewhat)	16 (29.6%)	1 (0.01%)
Placebo (Strongly)	23 (42.6%)	0 (0.0%)
	**54**	**128**

© 2023 Marcotte TD et al. *JAMA Psychiatry.*

eTable 4. Percentage of participants showing an FST clue at each time point by treatment arm among those classified as FST-impaired at the first FST evaluation (Time 1). Bold font indicates p-values <0.05.

**FST Clues**		*Time 1 (1h 10min)*	*Time 2 (2h 20min)*	*Time 3 (3h 10min)*	*Time 4 (4h 10min)*
**Walk and Turn Test**					
Instructions					
N (%) with clue	Placebo	11 (35.5%)	5 (16.1%)	2 (6.5%)	3 (9.7%)
	THC[1]	29 (30.5%)	15 (15.8%)	11 (11.6%)	6 (6.3%)
Change from Time 1*	Placebo	--	.08	**.005**	**.005**
	THC	--	**.001**	**<.001**	**<.001**
Differences in Change*	THC v Placebo	--	.76	.76	.76
Balance					
N (%) with clue	Placebo	12 (38.7%)	12 (38.7%)	9 (29.0%)	6 (19.4%)
	THC	49 (51.0%)	31 (32.3%)	21 (21.9%)	21 (21.9%)
Change from Time 1*	Placebo	--	>.99	.54	.08
	THC	--	**.001**	**<.001**	**<.001**
Differences in Change*	THC v Placebo	--	.17	.17	.50
Starts too soon					
N (%) with clue	Placebo[2]	2 (6.7%)	0 (0.0%)	1 (3.3%)	0 (0.0%)
	THC	8 (8.3%)	3 (3.1%)	1 (1.0%)	2 (2.1%)
Change from Time 1*	Placebo	--	n/a	n/a	n/a
	THC	--	.14	.11	.11
Differences in Change*	THC v Placebo	--	n/a	n/a	n/a
Stops when walking					
N (%) with clue	Placebo[2]	14 (46.7%)	9 (30.0%)	4 (13.3%)	4 (13.3%)
	THC	54 (56.2%)	38 (39.6%)	21 (21.9%)	12 (12.5%)
Change from Time 1*	Placebo	--	.12	**.008**	**.02**
	THC	--	**.01**	**<.001**	**<.001**
Differences in Change*	THC v Placebo	--	.94	.94	.94
Steps off line					
N (%) with clue	Placebo[2]	6 (20.0%)	7 (23.3%)	4 (13.3%)	3 (10.0%)
	THC	24 (35.4%)	24 (25.0%)	18 (18.8%)	20 (20.8%)
Change from Time 1*	Placebo	--	.76	.76	.76
	THC	--	**.03**	**.009**	**.01**
Differences in Change*	THC v Placebo	--	.93	.93	.93
Wrong number of steps					
N (%) with clue	Placebo[2]	6 (20.0%)	4 (13.3%)	5 (16.7%)	3 (10.0%)
	THC	24 (25.0%)	16 (16.7%)	12 (12.5%)	8 (8.3%)
Change from Time 1*	Placebo	--	.47	.65	.47
	THC	--	.06	**.02**	**.001**
Differences in Change*	THC v Placebo	--	.96	.73	.73
Misses heel to toe					
N (%) with clue	Placebo[2]	6 (20.0%)	8 (26.7%)	3 (10.0%)	3 (10.0%)
	THC	37 (38.5%)	28 (29.2%)	19 (19.8%)	15 (15.6%)
Change from Time 1*	Placebo	--	.41	.39	.39
	THC	--	.06	**<.001**	**<.001**
Differences in Change*	THC v Placebo	--	.35	.85	.85
Raises arm to balance					
N (%) with clue	Placebo[2]	17 (56.7%)	17 (56.7%)	11 (36.7%)	10 (33.3%)
	THC	58 (60.4%)	45 (46.9%)	34 (35.4%)	28 (29.2%)
Change from Time 1*	Placebo	--	>.99	**.04**	**.04**
	THC	--	.01	<.001	<.001
Differences in Change*	THC v Placebo	--	.51	.63	.63
Improper turn					
N (%) with clue	Placebo[2]	17 (56.7%)	14 (46.7%)	8 (26.7%)	9 (30.0%)
	THC	55 (57.3%)	42 (43.8%)	39 (40.6%)	29 (30.2%)
Change from Time 1*	Placebo	--	.31	**.02**	**.02**
	THC	--	**.008**	**.002**	**<.001**
Differences in Change*	THC v Placebo	--	.98	.76	.98
WAT ≥ 2					
N (%) with clue	Placebo[2]	25 (83.3%)	19 (63.3%)	12 (40.0%)	10 (33.3%)
	THC	82 (85.4%)	61 (63.5%)	50 (52.1%)	40 (41.7%)

© 2023 Marcotte TD et al. *JAMA Psychiatry.*

Change from Time 1*	Placebo	--	**.03**	**<.001**	**<.001**
	THC	--	**<.001**	**<.001**	**<.001**
Differences in Change*	THC v Placebo	--	.79	.79	.79
**One Leg Stand Test**					
Puts foot down					
N (%) with clue	Placebo[3]	8 (27.6%)	3 (10.3%)	3 (10.3%)	3 (10.3%)
	THC[4]	34 (37.4%)	21 (23.1%)	18 (19.8%)	20 (22.0%)
Change from Time 1*	Placebo	--	.06	.06	.06
	THC	--	**.006**	**.004**	**.005**
Differences in Change*	THC v Placebo	--	.65	.65	.65
Uses arms to balance					
N (%) with clue	Placebo[3]	14 (48.3%)	9 (31.0%)	10 (34.5%)	8 (27.6%)
	THC[5]	48 (52.2%)	39 (42.4%)	30 (32.6%)	25 (27.2%)
Change from Time 1*	Placebo	--	**.02**	**.03**	**.02**
	THC	--	.08	**<.001**	**<.001**
Differences in Change*	THC v Placebo	--	.68	.68	.68
Sways					
N (%) with clue	Placebo[2]	25 (83.3%)	18 (60.0%)	14 (46.7%)	14 (46.7%)
	THC[1]	88 (92.6%)	72 (75.8%)	54 (56.8%)	54 (56.8%)
Change from Time 1*	Placebo	--	**.004**	**<.001**	n/a
	THC	--	**<.001**	**<.001**	n/a
Differences in Change*	THC v Placebo	--	.74	.74	n/a
Hopping					
N (%) with clue	Placebo[3]	2 (6.9%)	1 (3.4%)	3 (10.3%)	1 (3.4%)
	THC[6]	7 (7.8%)	7 (7.8%)	7 (7.8%)	6 (6.7%)
Change from Time 1*	Placebo	--	.48	.57	.48
	THC	--	>.99	>.99	>.99
Differences in Change*	THC v Placebo	--	.63	.63	.63
OLS ≥ 2					
N (%) with clue	Placebo[3]	17 (58.6%)	10 (34.5%)	9 (31.0%)	8 (27.6%)
	THC[7]	57 (64.0%)	44 (49.4%)	35 (39.3%)	28 (31.5%)
Change from Time 1*	Placebo	--	**.004**	**.002**	**.002**
	THC	--	**.005**	**<.001**	**<.001**
Differences in Change*	THC v Placebo	--	.93	.93	.93
WAT ≥ 2 & OLS ≥ 2					
N (%) with clue	Placebo[8]	14 (50.0%)	6 (21.4%)	7 (25.0%)	4 (14.3%)
	THC[7]	50 (56.2%)	34 (38.2%)	26 (29.2%)	17 (19.1%)
Change from Time 1*	Placebo	--	**.003**	**.01**	**.002**
	THC	--	**.001**	**<.001**	**<.001**
Differences in Change*	THC v Placebo	--	.68	.95	.95
**Finger to Nose Test**					
Instructions					
N (%) with clue	Placebo	12 (38.7%)	7 (22.6%)	5 (16.1%)	4 (12.9%)
	THC	33 (34.4%)	13 (13.5%)	11 (11.5%)	7 (7.3%)
Change from Time 1*	Placebo	--	.09	**.05**	**.03**
	THC	--	**<.001**	**<.001**	**<.001**
Differences in Change*	THC v Placebo	--	.75	.75	.75
Incorrect sequence					
N (%) with clue	Placebo	7 (22.6%)	2 (6.5%)	3 (9.7%)	3 (9.7%)
	THC	19 (19.8%)	18 (18.8%)	15 (15.6%)	11 (11.5%)
Change from Time 1*	Placebo	--	.16	.16	.16
	THC	--	.84	.67	.35
Differences in Change*	THC v Placebo	--	.46	.48	.67
Uses pad rather than finger					
N (%) with clue	Placebo	20 (64.5%)	19 (61.3%)	17 (54.8%)	9 (29.0%)
	THC	63 (65.6%)	64 (66.7%)	40 (41.7%)	33 (34.4%)
Change from Time 1*	Placebo	--	.74	.54	**.003**
	THC	--	.84	**<.001**	**<.001**
Differences in Change*	THC v Placebo	--	.70	.70	.70
Leaves finger on nose					
N (%) with clue	Placebo	7 (22.6%)	2 (6.5%)	1 (3.2%)	2 (6.5%)
	THC	13 (13.5%)	5 (5.2%)	2 (2.1%)	0 (0.0%)

© 2023 Marcotte TD et al. *JAMA Psychiatry.*

Change from Time 1*	Placebo	--	.07	**.02**	n/a
	THC	--	.07	**.02**	n/a
Differences in Change*	THC v Placebo	--	.89	.90	n/a
Eyelid tremor					
N (%) with clue	Placebo	26 (83.9%)	18 (58.1%)	15 (48.4%)	11 (35.5%)
	THC	80 (83.3%)	68 (70.8%)	55 (57.3%)	41 (42.7%)
Change from Time 1*	Placebo	--	**.002**	**<.001**	**<.001**
	THC	--	**.004**	**<.001**	**<.001**
Differences in Change*	THC v Placebo	--	.56	.56	.56
Body tremor					
N (%) with clue	Placebo	4 (12.9%)	5 (16.1%)	3 (9.7%)	3 (9.7%)
	THC	28 (29.2%)	22 (22.9%)	13 (13.5%)	12 (12.5%)
Change from Time 1*	Placebo	--	.31	.31	.31
	THC	--	.05	**<.001**	**<.001**
Differences in Change*	THC v Placebo	--	.11	.11	.11
Sways					
N (%) with clue	Placebo	19 (61.3%)	14 (45.2%)	8 (25.8%)	4 (12.9%)
	THC[5]	67 (72.8%)	52 (56.5%)	30 (32.6%)	23 (25.0%)
Change from Time 1*	Placebo	--	**.02**	**<.001**	**<.001**
	THC	--	**.003**	**<.001**	**<.001**
Differences in Change*	THC v Placebo	--	.85	.85	.85
**Lack of Convergence Test**					
Instructions					
N (%) with clue	Placebo	0 (0.0%)	0 (0.0%)	0 (0.0%)	3 (9.7%)
	THC[9]	8 (8.5%)	4 (4.3%)	3 (3.2%)	0 (0.0%)
Change from Time 1*	Placebo	--	n/a	n/a	n/a
	THC	--	.21	.10	n/a
Differences in Change*	THC v Placebo	--	n/a	n/a	n/a
Eyes do not converge					
N (%) with clue	Placebo	24 (77.4%)	15 (48.4%)	12 (38.7%)	9 (29.0%)
	THC	75 (78.1%)	61 (63.5%)	38 (39.6%)	24 (25.0%)
Change from Time 1*	Placebo	--	**<.001**	**<.001**	**<.001**
	THC	--	**.002**	**<.001**	**<.001**
Differences in Change*	THC v Placebo	--	.61	.99	.99
**Modified Romberg Balance Test**					
Instructions					
N (%) with clue	Placebo	12 (38.7%)	5 (16.1%)	1 (3.2%)	2 (6.5%)
	THC[10]	30 (32.3%)	10 (10.8%)	12 (12.9%)	6 (6.5%)
Change from Time 1*	Placebo	--	**.02**	**.01**	**.02**
	THC	--	**<.001**	**<.001**	**<.001**
Differences in Change*	THC v Placebo	--	.77	.27	.77
Internal clock (unacceptable)					
N (%) with clue	Placebo	22 (71.0%)	19 (61.3%)	12 (38.7%)	16 (51.6%)
	THC[1]	41 (43.2%)	47 (49.5%)	35 (36.8%)	32 (33.7%)
Change from Time 1*	Placebo	--	.17	**.03**	.16
	THC	--	.27	.27	.24
Differences in Change*	THC v Placebo	--	.12	.12	.44
Eyelid tremors					
N (%) with clue	Placebo	27 (87.1%)	22 (71.0%)	18 (58.1%)	14 (45.2%)
	THC	81 (84.4%)	75 (78.1%)	60 (62.5%)	45 (46.9%)
Change from Time 1*	Placebo	--	.05	**.002**	**<.001**
	THC	--	.08	**<.001**	**<.001**
Differences in Change*	THC v Placebo	--	.63	.63	.63
Body tremors					
N (%) with clue	Placebo	11 (35.5%)	6 (19.4%)	4 (12.9%)	6 (19.4%)
	THC	25 (26.0%)	19 (19.8%)	14 (14.6%)	13 (13.5%)
Change from Time 1*	Placebo	--	**.02**	**.02**	**.02**
	THC	--	.08	**.006**	**.006**
Differences in Change*	THC v Placebo	--	.40	.40	.97
Sways					
N (%) with clue	Placebo	22 (71.0%)	19 (61.3%)	15 (48.4%)	12 (38.7%)
	THC[1]	74 (77.9%)	69 (72.6%)	51 (53.7%)	44 (46.3%)
Change from Time 1*	Placebo	--	.25	**.02**	**.001**

© 2023 Marcotte TD et al. *JAMA Psychiatry.*

	THC	--	.27	**<.001**	**<.001**
Differences in Change*	THC v Placebo	--	.91	.91	.91

* p-value, adjusted for multiple testing with false discovery rate (FDR) method
n/a = not available, cannot be reliably estimated due to insufficient data
Total n: 31 Placebo, 96 THC. Participants with one or more missing evaluations were excluded.
[1]n=95; [2]n=30; [3]n=29; [4]n=91; [5]n=92; [6]n=90; [7]n=89; [8]n=28; [9]n=94; [10]n=93.

eTable 4 Note

General estimating equations (GEE) method for binary outcomes was used to fit separate models regressing failed clue (yes/no) on THC treatment, time, and interaction. Time was treated as factor. Interaction p-values were used to test for differences in rate of change in proportion of participants failing each clue between Placebo and THC group. P-values were corrected for multiple testing with false discovery rate (FDR) method. Models were modified for four FSTs (WAT starts too soon, OLS sways, FTN leaves finger on nose, LOC instructions) by removing unusable data and adjusting the model equations accordingly. This was done, because reliable estimates could not be achieved from a full model either due to nobody showing that clue at one or more time points (i.e., zero frequency) or, in one instance, due to perfect collinearity (OLS sways at times 4 and 5). Abbreviation n/a is used to indicate p-values that could not be estimated.

© 2023 Marcotte TD et al. *JAMA Psychiatry.*

eTable 5. Association between demographic characteristics and officer-determined FST-impairment at 71 minutes post-smoking in the Placebo group (N=63).

	Officer determination at 71 minutes		Association with FST-Impairment #	
**Variable**	**Not Impaired**	**FST-Impaired**	**OR (95% CI)**	**P**
N	32	31		
Age, years; mean (SD)	27.9 (7.26)	28.4 (7.36)	1.01 (0.94, 1.08)	.79
Sex; N (%)				
Female	13 (40.6%)	18 (58.1%)	(reference)	--
Male	19 (59.4%)	13 (41.9%)	0.49 (0.18, 1.34)	.17
Education, years; mean (SD)	14.9 (2.03)	15.2 (1.85)	1.09 (0.84, 1.42)	.51
Race/Ethnicity; N (%)				
Non-white	21 (65.6%)	14 (45.2%)	(reference)	--
Non-Hispanic White	11 (34.4%)	17 (54.8%)	2.32 (0.85, 6.56)	.10
Miles driven previous year; median [IQR]	9,398 [5,560-15,100]	8,460 [5,040-11,280]	0.99 (0.96, 1.02) per 1000 miles	.55
Cannabis Use				
Current use; N (%)				
<4 times/week	19 (59.4%)	15 (48.4%)	(reference)	--
≥4 times/week	13 (40.6%)	16 (51.6%)	1.56 (0.58, 4.29)	.38
Grams used (last 30 days); median [IQR]	39 [9-131]	50 [18-162]	1.00 (0.97, 1.04) per 10 grams	.90
Days used (last 30 days); mean (SD)	16.0 (10)	18.0 (10)	1.01 (0.96, 1.07)	.59
Grams/day when using (last 30 days); median [IQR]	0.50 [0.25-0.90]	0.75 [0.38-1.00]	1.07 (0.59, 1.98)	.82
Days since last use; median [IQR]	3.00 [3.00-5.50]	3.00 [2.50-4.00]	1.05 (0.94, 1.20)	.38
Prescription medications with possible CNS effects	0 (0.0%)	3 (9.7%)[1]	7.98 (0.40, 161.2)	.18
Study measurements				
Blood THC concentration at 30 min (ng/mL)	0.79 (1.44)	1.64 (2.12)	1.31 (0.99, 1.83)	.06
Guess treatment correctly				
No	14 (45.2%)	18 (58.1%)	(reference)	--
Yes	17 (54.8%)	13 (41.9%)	0.59 (0.21, 1.62)	.31
Reported level of highness; median [IQR]	9.5 [0.0-20.5]	17 [2-30]	1.02 (0.99, 1.05)	.24
CDS at 30 minutes; mean (SD)	-0.32 (0.50)	-0.03 (0.67)	2.38 (0.99, 6.50)	.05
Impaired CDS at 30 min; N (%)	3 (10.0%)	6 (19.4%)	2.16 (0.51, 11.1)	.30

IQR = interquartile range; SD = standard deviation. # Estimated by logistic regression.
[1] Tricylic anti-depressant (n = 1); hydrocodone/acetaminophen (n = 1), oxycodone (n = 1)

© 2023 Marcotte TD et al. *JAMA Psychiatry.*

eTable 6. Univariable associations between FST items and being classified as FST-impaired by the officer in the Placebo group (N=63).

	Unimpaired (n=32)	FST-Impaired (n=31)	Association with Officer Determination of FST-Impairment				
**SFST Clues**	**n (%) with clue**	**n (%) with clue**	**OR**	**95% CI**	**P**	**Adj. 95% CI**	**Adj. P**
Walk and Turn Test							
Instructions	2 (6.2%)	11 (35.5%)	8.25	(1.65, 41.3)	.01	(0.85, 80.4)	.09
Balance	5 (15.6%)	12 (38.7%)	3.41	(1.03, 11.3)	.04	(0.79, 14.7)	.13
Starts too soon	1 (3.1%)	2 (6.7%)[1]	2.21	(0.19, 25.8)	.53	(0.19, 25.8)	.53
Stops when walking	6 (18.8%)	14 (46.7%)[1]	3.79	(1.21, 11.9)	.02	(0.86, 16.6)	.10
Steps off line	2 (6.2%)	6 (20.0%)[1]	3.75	(0.69, 20.3)	.12	(0.62, 22.7)	.17
Wrong number of steps	2 (6.2%)	6 (20.0%)[1]	3.75	(0.69, 20.3)	.12	(0.62, 22.7)	.17
Misses heel to toe	3 (9.4%)	6 (20.0%)[1]	2.42	(0.55, 10.7)	.25	(0.53, 11.1)	.28
Raises arm to balance	11 (34.4%)	17 (56.7%)[1]	2.50	(0.89, 6.97)	.08	(0.76, 8.17)	.17
Improper turn	12 (37.5%)	17 (56.7%)[1]	2.18	(0.79, 6.02)	.13	(0.75, 6.36)	.17
Total clues; median	**1.0**	**3.0**	**2.86**	**(1.63, 5.00)**	**<.001**	**(1.54, 5.31)**	**<.001**
WAT clues ≥ 2	**10 (31.2%)**	**25 (83.3%)[1]**	**11.0**	**(3.26, 37.1)**	**<.001**	--	--
One Leg Stand Test							
Puts foot down	2 (6.2%)	8 (26.7%)[1]	5.45	(1.05, 28.2)	.04	(0.95, 31.2)	.06
**Uses arms to balance**	**6 (18.8%)**	**15 (50.0%)[1]**	**4.33**	**(1.39, 13.6)**	**.01**	**(1.18, 16.0)**	**.02**
**Sways**	**9 (28.1%)**	**26 (83.9%)**	**13.3**	**(3.89, 45.4)**	**<.001**	**(2.78, 63.6)**	**<.001**
Hopping	0 (0.0%)	2 (6.7%)[1]	5.70	(0.44, 799)	.20	(0.44, 799)	.20
Total clues; median	**0.0**	**2.0**	**3.95**	**(1.96, 7.93)**	**<.001**	**(1.72, 9.04)**	**<.001**
OLS clues ≥ 2	**5 (15.6%)**	**18 (60.0%)[1]**	**8.10**	**(2.44, 26.9)**	**<.001**	--	--
**WAT≥ 2 & OLS≥ 2**	**2 (6.2%)**	**15 (51.7%)[2]**	**16.1**	**(3.23, 80.1)**	**<.001**	--	--
Finger to Nose Test							
**Instructions**	**1 (3.1%)**	**12 (38.7%)**	**19.6**	**(2.35, 163)**	**.006**	**(1.39, 277)**	**.02**
Incorrect sequence	2 (6.2%)	7 (22.6%)	4.37	(0.83, 23.0)	.08	(0.79, 24.3)	.10
Uses pad rather than finger	12 (37.5%)	20 (64.5%)	3.03	(1.09, 8.46)	.03	(0.95, 9.66)	.06
Leaves finger on nose	1 (3.1%)	7 (22.6%)	9.04	(1.04, 78.6)	.05	(0.89, 91.7)	.06
Eyelid tremor	19 (59.4%)	26 (83.9%)	3.56	(1.08, 11.7)	.04	(0.94, 13.4)	.06
Body tremor	2 (6.2%)	4 (12.9%)	2.22	(0.38, 13.1)	.38	(0.38, 13.1)	.38
**Sways**	**3 (9.4%)**	**19 (61.3%)**	**15.3**	**(3.81, 61.5)**	**<.001**	**(2.27, 103)**	**.001**
Total clues; median	**1.0**	**3.0**	**4.10**	**(2.10, 8.00)**	**<.001**	**(1.70, 9.86)**	**<.001**
Lack of Convergence							
Instructions	3 (9.4%)	0 (0.0%)	0.13	(0.001, 1.47)	.11	(0.001, 1.47)	.11
**Eyes do not converge**	**7 (21.9%)**	**24 (77.4%)**	**12.2**	**(3.73, 40.2)**	**<.001**	**(3.15, 47.6)**	**<.001**
Pupillary diameter, average (mm) [#]; median	4.5	5.5	1.55	(0.95, 2.50)	.08	--	--
Total clues; median	**0.0**	**1.0**	**7.54**	**(2.45, 23.3)**	**<.001**	**(2.32, 24.5)**	**.001**
Modified Romberg Balance Test							
Instructions	8 (25.0%)	12 (38.7%)	1.89	(0.64, 5.57)	.25	(0.64, 5.57)	.25
Internal clock (seconds)[#]; median	32	38	1.02	(0.99, 1.06)	.18	--	--
**Internal clock (not acceptable)**	**12 (37.5%)**	**22 (71.0%)**	**4.07**	**(1.42, 11.7)**	**.009**	**(1.02, 16.3)**	**.05**
Eyelid tremors	23 (71.9%)	27 (87.1%)	2.64	(0.72, 9.72)	.14	(0.62, 11.2)	.24
Body tremors	6 (18.8%)	11 (35.5%)	2.38	(0.75, 7.55)	.14	(0.66, 8.60)	.24
Sways	18 (56.2%)	22 (71.0%)	1.90	(0.67, 5.40)	.23	(0.66, 5.49)	.25
Total clues; median	**2.0**	**3.0**	**2.31**	**(1.33, 3.99)**	**.003**	**(1.33, 3.99)**	**.003**
Total Clues; median	**5.0**	**11.0**	**2.53**	**(1.59, 4.04)**	**<.001**	**(1.59, 4.04)**	**<.001**

[1]n=30; [2]n=29

© 2023 Marcotte TD et al. *JAMA Psychiatry.*

eAppendix

Driving simulations

Driving simulations: Driving simulations were presented on a STISIM M300WS-Console Driving Simulator System (Systems Technology, Inc; Hawthorne, CA) consisting of 3-screen, wide field-of-view monitors, steering wheel, and accelerator and brake pedals, and programmed using STISIM Drive v3.14[1]. The fully interactive simulation included routine and non-routine events throughout the drive. This included driving within residential, commercial and highway sections. During these drives participants encountered intersections, moving traffic, pedestrians, stop signs, and other challenges a driver may encounter on a road. Each simulation included intersections in which the participant would encounter the “yellow light dilemma”, wherein individuals need to respond to a yellow light (timed to be consistent with California Law) and decide whether to stop or continue on, possibly risking running a red light; scenarios in which the participant is to merge with highway traffic, and quickly exit the roadway; make left turns in front of on-coming traffic, and other decision-making situations. Each drive also included a pre-determined crash avoidance scenario in which the participant drives down a visually complex roadway (moving cars, pedestrians) and encounters the sudden appearance of a pedestrian, or car pulling out, in the roadway. While all participants encountered the same scenarios, these were in part free drives in that the participant could adjust their speed, choose lane positions, etc. as desired. The simulations covered approximately 10.5 miles and took approximately 25 minutes to complete.

Importantly, within the context of this “normal” drive we included controlled scenarios that have previously been shown to be sensitive to acute cannabis use and other impairment-causing conditions at specified points in the simulation:

<u>Modified Surrogate Reference Test (mSuRT)</u>. This is a divided attention task, modified from the Surrogate Reference Task[2], and developed in collaboration with colleagues at Brainbaseline®. Upon initiation of the task (a phone would ring), participants were asked to view an iPad, off to the side of the simulator monitors (Figure A). The iPad shows a pattern of random, hollow circles, with one of the circles being different (larger) than the others. The participant’s task is to locate and touch the larger circle. The level of difficulty is varied by changing the ratio of the size of the distractor circles and target circles. Importantly, the participant is instructed to also maintain the appropriate speed (65 mph) and maintain the correct position in the center of the lane. In order for this to yield performance under a controlled condition (thus facilitating group analyses), no other traffic is on the road during this time. There are 16 trials each within the *easy* and *hard* versions. The next stimulus appears immediately after the participant’s response. The secondary task (mSuRT) was presented at the same pre-determined location for all participants. They were expected to complete the task when it started. It takes approximately 60 seconds to complete the easy task, and 60-90 seconds for the hard task, depending upon how rapidly the participant responded to each stimulus.

This is a measure of performance under high cognitive load and controlled processing, in that participants must divide their attention among three stimuli (roadway, speedometer, and events in the periphery), and is reflective of the workload generated by a real task (e.g., a GPS system). While we considered using more face valid interfaces (such as an iPhone) and tasks (e.g., identifying musical tracks), this surrogate or structured task allow us to look at changes in attention in a more controlled fashion (not affected by familiarity with interface, reading speed, etc.).

© 2023 Marcotte TD et al. *JAMA Psychiatry.*

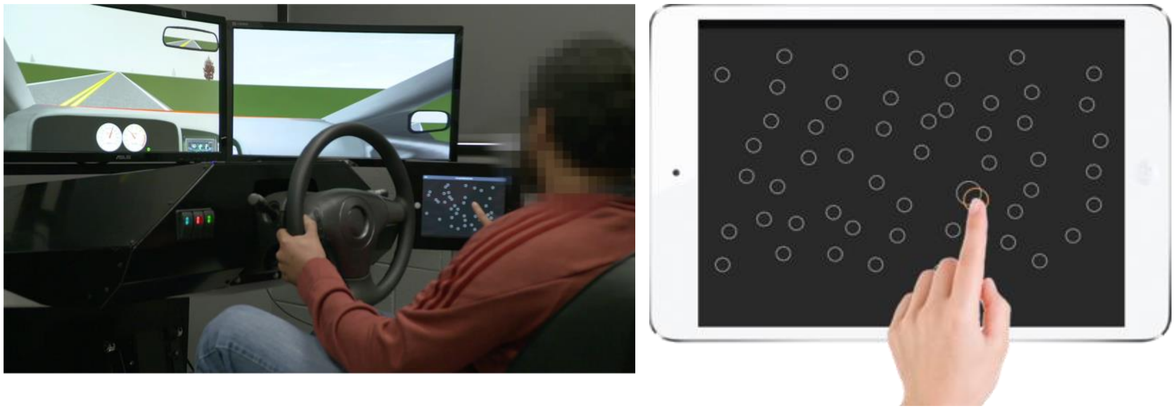

Figure A. Modified Surrogate Reference Task (mSuRT)

Car Following. Once during each simulation, at a specified location the participants were required to adjust their speed to a lead car that speeds up and slows down according to a sinusoidal wave. The primary outcome is the coherence between the participant and lead cars (a general correlation [0–1] of the participant's ability to accurately track the speed variations of the lead car). Time delay (or the reaction time to changes in the lead car's speed) and distance from the lead car were also variables of interest.

Note that individuals desiring greater details regarding the simulations may contact the lead investigator at tmarcotte@health.ucsd.edu.

Composite Drive Score. Driving simulators bring with them the ability to collect massive amounts of data. In some cases, even targeted scenarios have multiple outcomes of interest. This comes at a cost, though, in that it is not always clear regarding whether an individual, overall, evidenced a decline in driving performance. To address this, in addition to analyzing individual outcome variables we developed a Composite Drive Score that incorporates the key variables from the two scenarios above and combined them in a manner to create a single score. We then created a baseline anchor for performance based upon the performance of all 191 participants during their pre-smoking drive. All subsequent Composite Drive Scores used this as the basis for developing the score at each timepoint, thus facilitating analysis of change in performance from pre-treatment.

In order to accomplish this, z-scores were established based upon the pre-smoking simulator performance, using the mean and standard deviation on each score for all 191 participants. Z-scores for each participant were calculated by subtracting the group mean score from the participant's score and dividing that by the group standard deviation (so that, in the end, at the pre-smoke driving the Composite Drive Score for the entire sample had a mean z-score of 0, with a standard deviation of 1). Higher z-scores at each timepoint indicate worse performance (variables that went in the opposite direction were reflected in order to have all variables have the same direction). The Composite Drive Score was comprised of the following variables: mSuRT task (SDLP, Speed Deviation, correct hits on SuRT) and Car Following (coherence).

The validity of SDLP and Car Following tasks in detecting declines in performance relating to cannabis and other substances has been widely reported[3-6; 7,8; 9]. Developing a

© 2023 Marcotte TD et al. *JAMA Psychiatry.*

composite score, used frequently in other types of behavioral studies, overcomes one limitation in cannabis/driving studies noted in a recent comprehensive review– an emphasis on multiple dependent variables[10]. A similar approach has been used by others[11,12], demonstrating sensitivity to cannabis consumption and aging. This is the first time incorporating these specific measures from this simulator into a composite score.

Determination of Impairment.
To determine impairment on the simulator, based upon previous methodological work examining cutpoints for cognitive measures in relation to brain function[13-15], driving impairment at 30 min was classified using a cutpoint for impairment approximating the upper 15th percentile (higher CDS scores associated with worse performance) in the Placebo group. This cutpoint was then also applied to the THC group.

Randomization/Blinding

Treatment groups were assigned using permuted blocks randomization with stratification by prior cannabis exposure (frequent user [≥4x per week] versus occasional user [≤4x per week]). The allocation schedule was kept in the UCSD Research Pharmacy, which prepared the cannabis material, and the schedule was concealed from other study personnel. Participants and assessors were blinded to group assignments.

Power/sample size

The overall study sample size was determined based upon driving simulator outcomes. In a previous study using a single monitor and less challenging divided attention task (stimuli would appear on the screen itself) we found that participants who smoked cannabis cigarettes with 4% THC evidenced effect sizes between 0.36 and 0.47 when comparing changes in SDLP between placebo and active THC at 2 to 3 hours post-smoking[19]. For power calculations for this study, it was assumed that the placebo group will show minimal changes in CDS over time and that the 13.4% THC group will show a worsening in CDS immediately after smoking cannabis with a gradual return to expected CDS levels afterwards. Cohen's d was used as an estimate for the effect size for measuring the difference in changes in CDS from baseline (pre-cannabis) to the time point with the assumed largest differences between the two groups. Under these assumptions, power for finding a significant difference in changes in CDS between the 13.4% THC group (n = 60) and the placebo (n = 60) was estimated using 1000 simulations, which showed 80% power to detect Cohen's d=0.33 or larger with significance level α=0.05.

Adverse events

There were no serious adverse events recorded in the study. A total of twenty-four participants (12% out of the 199 enrolled) reported experiencing one to five adverse events for a total of 46 events, including 44 mild (grade 1) and 2 moderate (grade 2). During the screening visit, 6 adverse events, consisting of dizziness (1), nausea (3), and vomiting (2), were reported by 4 participants. Since smoking did not occur, these were associated with driving simulator-induced motion sickness.

At the primary study visit, 22 participants (Placebo: n=2, 5.9% THC: n=15, 13.4% THC: n=5; Fisher's Exact test p=.002) reported 40 adverse events (Placebo: 2, 5.9% THC: 30, 13.4% THC: 8). Most common symptoms were abnormal heart rate (13), dizziness (6), changes in blood pressure (4 decreased, 2 increased) and nausea (3). Other reported symptoms included anxiety (2), discomfort (2), sweats (2), and one of each for cough, fainting, fever, headache,

© 2023 Marcotte TD et al. *JAMA Psychiatry.*

numbness in the arm, and swollen arm. Other than one participant who withdrew due to anxiety, all AEs resolved and participants continued with their visit.

© 2023 Marcotte TD et al. *JAMA Psychiatry.*

eReferences

1. Rosenthal TJ. *STISIM Drive*. Systems Technology, Inc.; 2017.
2. Ostlund J, Nilsson L, Tomros J, Forsman A. *Calibration tasks for methods which assess demand due to the use of invehicle systems*. 2006.
3. Bosker WM, Kuypers KP, Theunissen EL, et al. Medicinal Delta(9) -tetrahydrocannabinol (dronabinol) impairs on-the-road driving performance of occasional and heavy cannabis users but is not detected in Standard Field Sobriety Tests. *Addiction*. Oct 2012;107(10):1837-44. doi:10.1111/j.1360-0443.2012.03928.x
4. Hartman RL, Brown TL, Milavetz G, et al. Cannabis effects on driving lateral control with and without alcohol. *Drug Alcohol Depend*. Sep 1 2015;154:25-37. doi:10.1016/j.drugalcdep.2015.06.015
5. Arkell TR, Lintzeris N, Kevin RC, et al. Cannabidiol (CBD) content in vaporized cannabis does not prevent tetrahydrocannabinol (THC)-induced impairment of driving and cognition. *Psychopharmacology*. 2019;236(9):2713-2724.
6. Ramaekers JG, Robbe HW, O'Hanlon JF. Marijuana, alcohol and actual driving performance. *Hum Psychopharmacol*. Oct 2000;15(7):551-558. doi:10.1002/1099-1077(200010)15:7<551::AID-HUP236>3.0.CO;2-P
7. Lenne MG, Dietze PM, Triggs TJ, Walmsley S, Murphy B, Redman JR. The effects of cannabis and alcohol on simulated arterial driving: Influences of driving experience and task demand. *Accid Anal Prev*. May 2010;42(3):859-66. doi:10.1016/j.aap.2009.04.021
8. Brown TL, Richard C, Meghdadi A, et al. EEG biomarkers acquired during a short, straight-line simulated drive to predict impairment from cannabis intoxication. *Traffic Inj Prev*. Sep 25 2020:1-5. doi:10.1080/15389588.2020.1814957
9. Hartman RL, Brown TL, Milavetz G, et al. Cannabis effects on driving longitudinal control with and without alcohol. *J Appl Toxicol*. Nov 2016;36(11):1418-29. doi:10.1002/jat.3295
10. Alvarez L, Colonna R, Kim S, et al. Young and under the influence: A systematic literature review of the impact of cannabis on the driving performance of youth. *Accid Anal Prev*. Mar 2021;151:105961. doi:10.1016/j.aap.2020.105961
11. Ortiz-Peregrina S, Ortiz C, Castro-Torres JJ, Jimenez JR, Anera RG. Effects of Smoking Cannabis on Visual Function and Driving Performance. A Driving-Simulator Based Study. *Int J Environ Res Public Health*. Dec 3 2020;17(23)doi:10.3390/ijerph17239033
12. Wood JM. Age and visual impairment decrease driving performance as measured on a closed-road circuit. *Hum Factors*. Fall 2002;44(3):482-94. doi:10.1518/0018720024497664
13. Tulsky DS, Saklofske DH, Chelune GJ, et al, eds. *Clinical interpretation of the WAIS-III and WMS-III*. Academic Press; 2003.
14. Heaton RK, Miller SW, Taylor MJ, Grant I. *Revised Comprehensive norms for an expanded Halstead-Reitan Battery: Demographically adjusted neuropsychological norms for African American and Caucasian adults*. Psychological Assessment Resources, Inc.; 2004.
15. Campbell LM, Fennema-Notestine C, Saloner R, et al. Use of Neuroimaging to Inform Optimal Neurocognitive Criteria for Detecting HIV-Associated Brain Abnormalities. *J Int Neuropsychol Soc*. Feb 2020;26(2):147-162. doi:10.1017/S1355617719000985

© 2023 Marcotte TD et al. *JAMA Psychiatry.*
