# Supplementary material for: Evaluation of Field Sobriety Tests for Identifying Drivers Under the Influence of Cannabis: A Randomized Clinical Trial
Source: JAMA Psychiatry. 2023 Aug 2;80(9):914–23. doi: 10.1001/jamapsychiatry.2023.2345 (PMC10398547; doi:10.1001/jamapsychiatry.2023.2345)
Supplement: Supplement 3. — Data Sharing Statement [file jamapsychiatry-e232345-s003.pdf]

Data Sharing Statement

Marcotte. Evaluation of Field Sobriety Tests for Identifying Drivers Under the Influence of Cannabis. *JAMA Psychiatry*. Published August 02, 2023.
doi:10.1001/jamapsychiatry.2023.2345

Data
Data available: Yes
Data types: Deidentified participant data, Data dictionary
How to access data: cmcr@ucsd.edu
When available: With publication

Supporting Documents
Document types: None

Additional Information
Who can access the data: Approved researchers
Types of analyses: Meta-analysis
Mechanisms of data availability: With signed data use agreement
